# Quantum-chemical, NMR, FT IR, and ESI MS studies of complexes of colchicine with Zn(II)

**DOI:** 10.1007/s00894-017-3306-z

**Published:** 2017-03-20

**Authors:** Wojciech Jankowski, Joanna Kurek, Piotr Barczyński, Marcin Hoffmann

**Affiliations:** Faculty of Chemistry, Adam Mickiewicz University in Poznan, ul. Umultowska 89b, 61-614 Poznań, Poland

**Keywords:** Colchicine, Complexes of colchicine with metal cations, Quantum chemical calculations, DFT, ESI MS, NMR, FT IR

## Abstract

Colchicine is a tropolone alkaloid from *Colchicinum autumnale*. It shows antifibrotic, antimitotic, and anti-inflammatory activities, and is used to treat gout and Mediterranean fever. In this work, complexes of colchicine with zinc(II) nitrate were synthesized and investigated using DFT, ^1^H and ^13^C NMR, FT IR, and ESI MS. The counterpoise-corrected and uncorrected interaction energies of these complexes were calculated. We also calculated their ^1^H, ^13^C NMR, and IR spectra and compared them with the corresponding experimentally obtained data. According to the ESI MS mass spectra, colchicine forms stable complexes with zinc(II) nitrate that have various stoichiometries: 2:1, 1:1:1, and 2:1:1 with respect to colchichine, Zn(II), and nitrate ion. All of the complexes were investigated using the quantum theory of atoms in molecules (QTAIM). The calculated and the measured spectra showed differences before and after the complexation process. Calculated electron densities and bond critical points indicated the presence of bonds between the ligands and the central cation in the investigated complexes that satisfied the quantum theory of atoms in molecules.

**Electronic supplementary material:**

The online version of this article (doi:10.1007/s00894-017-3306-z) contains supplementary material, which is available to authorized users.

**Graphical Abstract:**
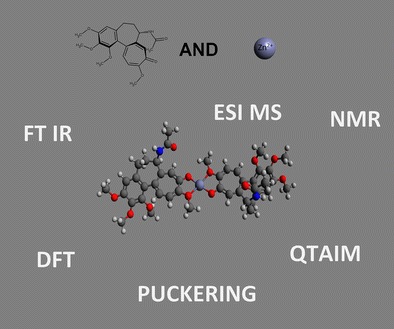
DFT, NMR, FT IR, ESI MS, QTAIM and puckering studies of complexes of colchicine with Zn(II).

## Introduction

**Fig. 1:**
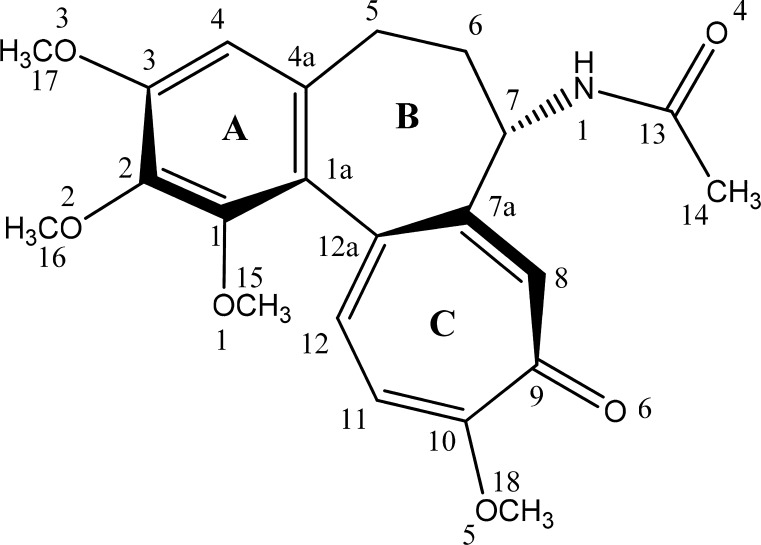
Numbering scheme used for carbon and oxygen atoms in colchicine **1**

Colchicine (Fig. 1) is a tropolone alkaloid from *Colchicum autumnale*. It naturally occurs as a neutral molecule; it does not form salts because of its very low basicity. This alkaloid possesses antimitotic, antifibrotic, anti-inflammatory activities. For instance, it can efficiently alleviate the symptoms of gout when applied in the early phase because of its anti-inflammatory properties [1–3], and it is a potent antimitotic agent, showing anticarcinogenic activity [4, 5]. As also seen for other alkaloids, colchicine can block or activate specific receptors (for example P2X7 and P2X2 [6]) or ion channels (for example the TRAAK [7] potassium channel) in living organisms. Its activity depends on its ability to form noncovalent complexes with macromolecules such as tubulin in microtubules.

There are only a few studies of the formation of complexes between colchicines and cations [8]. In 1998, Mackay et al. obtained hydrated crystals of copper(II) colchiceine (10-demethoxy-10-hydroxycolchicine) [9]. In a previous work, we reported the coordination of colchicine to iodides and perchlorates with monovalent metal ions (lithium, sodium, and potassium salts) [10]. Recent ab initio studies of the Na^+^–colchicine complex showed that its most stable geometry is obtained when the Na^+^ ion is located above the methoxytropolonic ring (Fig. 1, ring C) [11].

Complexes with zinc are interesting because zinc cations are biologically important for plants and animals. Zinc is responsible for a number of different functions in the human body because it is associated with various biomolecules (for example carbonic anhydrase, thermolysin, 5-aminolevulinate dehydratase) [12, 13]. It is the second most abundant metal (after iron) in the human body; it is essential for growth and development and plays important roles in various biological systems [14]. Zinc fingers play a crucial role in DNA base sequence recognition during the replication and transcription of DNA. Approximately 10% of all proteins in the human body can bind zinc, and hundreds of them can transport it [15, 16]. Zinc also plays a role in the brain. It has a specific neuromodulatory role in addition to its other cellular functions [17, 18].

From a practical point of view, the process of complexation can be useful for isolating colchicine from plant extracts or for effectively separating (complexed) colchicine from mixtures in HPLC methods. Indeed, colchicine can form stable complexes with zinc cations in human body fluids following the administration of colchicine as a drug during antigout therapy (i.e., patients take pills in which the active substance is colchicine).

Although colchicine is a very important commercially available alkaloid, its complexes (except for those with lithium, sodium, and potassium [10]) and the complexes of colchicine derivatives have generally not been thoroughly characterized. For instance, the process for the complexation of colchicine with zinc nitrate has not yet been studied. This fact prompted us to synthesize and examine complexes of colchicine with zinc(II) nitrate experimentally and computationally to find out if colchicine is likely to interact with the Zn(II) cation in the human body.

## Experimental methods

### Materials

Colchicine **1** was obtained from AppliChem (Darmstadt, Germany). The natural isomer of colchicine (−)-(a*R*,7*S*)-colchicine was used for complexation. The salt Zn(NO_3_)_2_ was obtained from Sigma–Aldrich (St. Louis, MO, USA) and used without any further purification. Solvents used for the synthesis were obtained from Sigma–Aldrich and purified by standard methods.

### Synthesis of the 1:1 complex of colchicine with zinc(II) nitrate

The 1:1 complex of zinc(II) nitrate with colchicine [Zn(C_22_H_25_NO_6_)(NO_3_)_2_] was obtained by dissolving the respective salt (76 mg, 0.25 mM) and colchicine (100 mg, 0.25 mM) in the ratio 1:1 in 10 mL of methanol. This mixture was stirred for 24 h at room temperature. The solution was evaporated until the product began to precipitate. The resulting precipitate was filtered off and recrystallized from methanol, and this colchicine complex was studied by spectral analysis using ESI MS, ^1^H and ^13^C NMR, and FT IR as well as theoretically. The carbon atom numbering scheme used for colchicine **1** is shown in Fig. 1.

### Measurements

ESI (electrospray ionization) mass spectra were recorded on a Waters/Micromass (Manchester, UK) ZQ mass spectrometer equipped with a Harvard Apparatus (Holliston, MA, USA) syringe pump. All samples were prepared in acetonitrile. The measurements were performed on solutions of colchicine (5 × 10^−5^ mol dm^−3^) with Zn(II) nitrate (2.5 × 10^−4^ mol dm^−3^). The sample was infused into the ESI source using a Harvard Apparatus pump at a flow rate of 20 l min^−1^. The ESI source potentials were: capillary 3 kV, lens 0.5 kV, extractor 4 V. Standard ESI mass spectra were recorded at 30 V. The source temperature was 120 °C and the desolvation temperature was 300 °C. Nitrogen was used as the nebulizing and desolvation gas at flow rates of 100 and 300 dm^3^ h^−1^, respectively. Mass spectra were acquired in the positive ion detection mode with unit mass resolution in steps of 1 *m*/*z* unit. The mass range applied in the ESI experiments was from* m*/*z* = 100 to* m*/*z *= 1400. Elemental analysis (% C, N, H) was carried out by means of a Vario EL III element analyzer (Elementar Analysensysteme GmbH, Langenselbold, Germany). Melting point data were obtained with a BÜCHI Labortechnik AG (Flawil, Switzerland) SMP-20 and a Mel-Temp II apparatus (Laboratory Devices Inc., Holliston, MA, USA).

NMR spectra of colchicine and its complex with zinc(II) nitrate (0.07 mol L^−1^) were recorded in CD_3_CN solution using a Varian (Palo Alto, CA, USA) Gemini 300 MHz spectrometer. All spectra were locked to the deuterium resonance of CD_3_CN. ^1^H NMR measurements in CD_3_CN were carried out at an operating frequency of 300.075 MHz; flip angle, pw = 45°; spectral width, sw = 4500 Hz; acquisition time, at = 2.0 s; relaxation delay,* d*_1_ = 1.0 s;* T* = 293.0 K, and using TMS as the internal standard. No window function or zero filling was used. The digital resolution was 0.2 Hz per point. The error in the chemical shift value was 0.01 ppm. ^13^C NMR spectra were recorded at an operating frequency of 75.454 MHz; pw = 60°; sw = 19000 Hz; at = 1.8 s;* d*_1_ = 1.0 s;* T* = 293.0 K, and using TMS as the internal standard. Line-broadening parameters were 0.5 or 1 Hz. The error in the chemical shift value was 0.01 ppm. The ^1^H and ^13^C NMR signals were assigned for each species using one- or two-dimensional (COSY, HETCOR, HMBC) spectra. FT IR spectra of colchicine and its complex with zinc nitrate (0.07 mol dm^−3^) were recorded in the mid-infrared region in KBr pellets, nujol, and CD_3_CN using a Bruker (Karlsruhe, Germany) IFS 113v spectrometer equipped with a DTGS detector; resolution 2 cm^−1^, NSS = 125. A cell with Si windows and wedge-shaped layers was used to avoid interference (mean layer thickness: 170 μm). Each FT IR spectrum was measured by acquiring 64 scans. All manipulation of the substances was performed in a carefully dried and CO_2_-free glove box.

### Theoretical calculations

All of the structures needed for the theoretical calculations were obtained from the known crystal structure of colchicine dehydrate (COLCDH) [19]. Energy calculations were performed using DFT at the M06/SDD level of theory [20, 21], which was selected on the basis of the results from the extensive comparative studies of Zhao and Truhlar [20] and because it is recommended for calculations of compounds containing metal atoms [13, 20–22]. Partial atomic charges were calculated at the same level of theory. In our studies, we utilized Mulliken [23] point charges. We also calculated the Wiberg bond indices [24] by natural bond orbital (NBO) analysis [25, 26] for the bonds between the ligands and the central zinc(II) cation in all of the investigated complexes. The counterpoise correction [27, 28] was calculated to assess the basis set superposition error (BSSE). IR spectra were calculated at the same level of theory as that used to perform the geometry optimizations. NMR spectra were calculated using the M06 functional with the SDD and pcS-2 basis sets [29] (the latter is recommended for use when calculating NMR shifts for complexes with organic molecules [30]) using the usual GIAO (gauge-independent atomic orbital) method [31]. Energy, NMR, and IR calculations were performed in the presence of solvent using the PCM model [32]. The M06/6-31+G(d,p) [33] level of theory was used to calculate bond critical points. That allowed us to determine whether colchicine forms bonds with the zinc(II) cation that satisfy the QTAIM (quantum theory of atoms in molecules) [34]. All quantum-mechanical calculations were performed in Gaussian 09 [35].

The conformation of the seven-membered ring in colchicine (ring B; see Fig. 1) was examined as described by Cremer and Pople [36], Boessenkool and Boyens [37], and Bocian et al. [38]. Four conformational parameters of the seven-membered ring were calculated: two puckering amplitudes* q*_2_ and* q*_3_ and two phase angles* φ*_2_ and* φ*_3_. Those parameters were calculated according to the following equations:

1 $$ {\rho}_m \cos {\upvarphi}_m={\left(\frac{2}{N}\right)}^{0.5}{\displaystyle \sum_{j=1}^N{z}_j \cos \left[\frac{2\pi m\left( j-1\right)}{N}\right]} $$

2 $$ {\rho}_m sin{\upvarphi}_m={\left(\frac{2}{N}\right)}^{0.5}{\displaystyle \sum_{j=1}^N{z}_j sin\left[\frac{2\pi m\left( j-1\right)}{N}\right]}, $$

where:

*m*: is 2 or 3
*ρ*_*m*_: is a puckering amplitude
*φ*_*m*_: is a phase angle
*N*: is the number of atoms in the ring
*z*_*j*_: is the displacement from the main plane, calculated from the position vector of atom* j*.

**Fig. 2:**
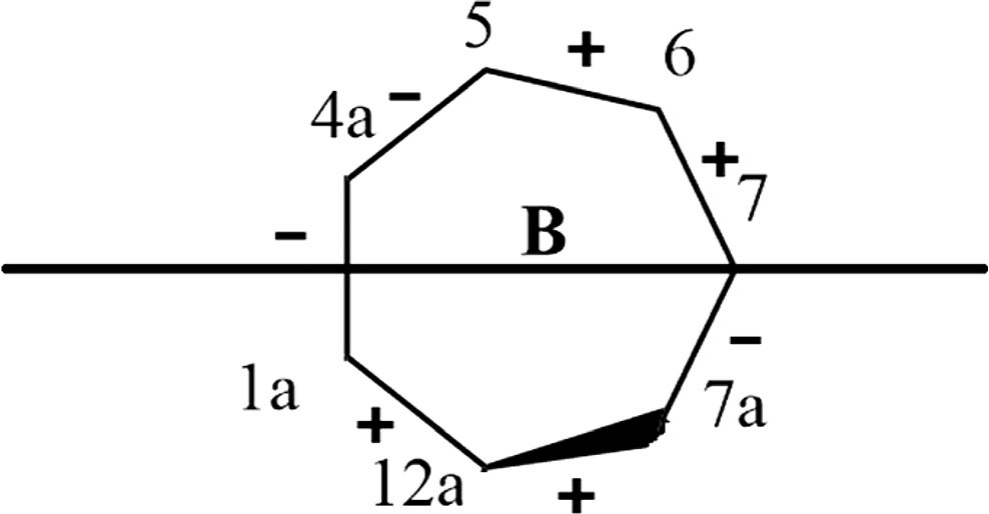
Atom order used for conformational analysis; the signs of the dihedral angles for the most energetically favored structures are also shown

As defined by Boessenkool and Boyens [37], each ring conformation was categorized as either a chair, twisted chair, boat, twisted boat, sofa, or twisted sofa. All conformational parameters of the seven-membered ring were calculated starting from carbon atom C7 (see Fig. 1) and moving clockwise, i.e., in the order C7-C7a-C12a-C1a-C4a-C5-C6 (see Fig. 2). The C7 atom was chosen as the starting point because it was the atom that was furthest out of plane in the majority of the most energetically favored structures.

## Results and discussion

### ESI MS measurements

**Fig. 3:**
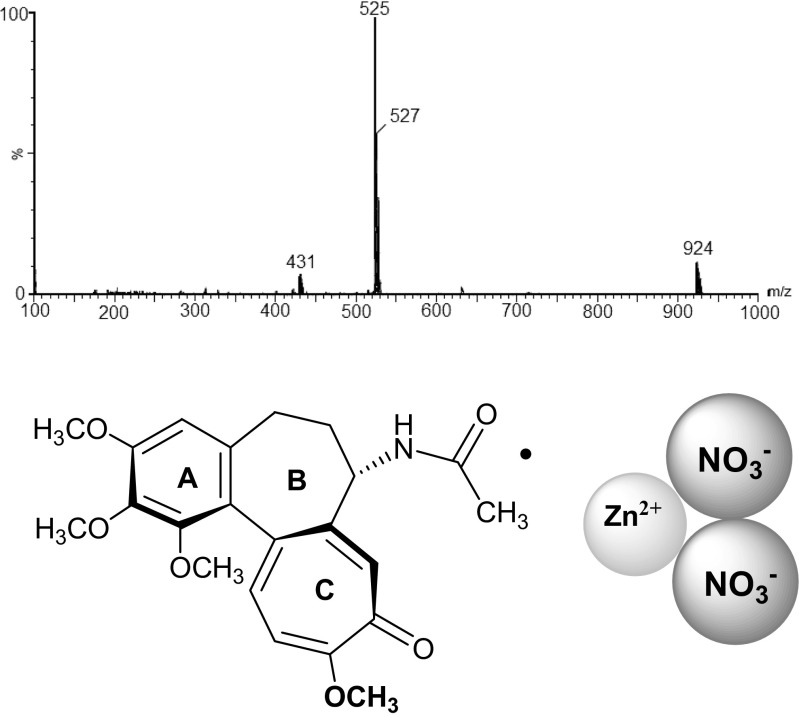
The ESI mass spectra (obtained in ES^+^ mode) of the complexes of colchicine with zinc(II) nitrate (i.e.,** 1**–Zn), as measured at cv = 30 V, as well as a diagram of the structure of the colchicine complex with Zn(NO_3_)_2_

Only three signals (at* m*/*z* = 431, 525, and 924) were observed in the ESI mass spectra obtained after complexation, which were assigned to colchicine–Zn(II) and colchicine–Zn(II)–NO_3_ complexes. The* m*/*z* signals in the ESI mass spectra of the complex formed between colchicine and zinc(II) nitrate at a cone voltage of 30 V are given in Table 1 and are shown in Fig. 3. The signal at* m*/*z* = 431 was assigned to a complex with a stoichiometry of 2:1 (i.e., two colchicine molecules and one divalent metal cation). The signal at* m*/*z* = 525 was assigned to a 1:1:1 complex [colchicine + Zn^2+^ + NO_3_^−^]^+^. The third characteristic signal, at* m*/*z* = 924, was assigned to a 2:1:1 complex [2 × colchicine + Zn^2+^ + NO_3_^−^]^+^. For the full ESI mass spectral data, see Fig. S1 in the “Electronic supplementary material” (ESM).

**Table 1:** Main peaks in the ESI mass spectra (obtained in ES^+^ mode) of the complexes of colchicine with zinc(II) nitrate, measured at cv = 30 V

Complex	*m*/*z*		
	2:1 [2 x **1** + Zn^2+^]	1:1:1 [**1** + Zn^2+^ + NO_3_^−^]	2:1:1 [2 × **1** + Zn^2+^ + NO_3_^−^]
Colchicine–Zn	431	525	924

**1** is the colchicine molecule

### Theoretical studies

The ESI MS studies showed that colchicine can form stable complexes with different stoichiometries (2:1, 1:1:1, and 2:1:1) which may or may not contain a nitrate anion. Nine different interaction schemes of colchicine complexes with zinc nitrate based on previously described possible interactions [39] were subjected to further computational investigation.

**Fig. 4:**
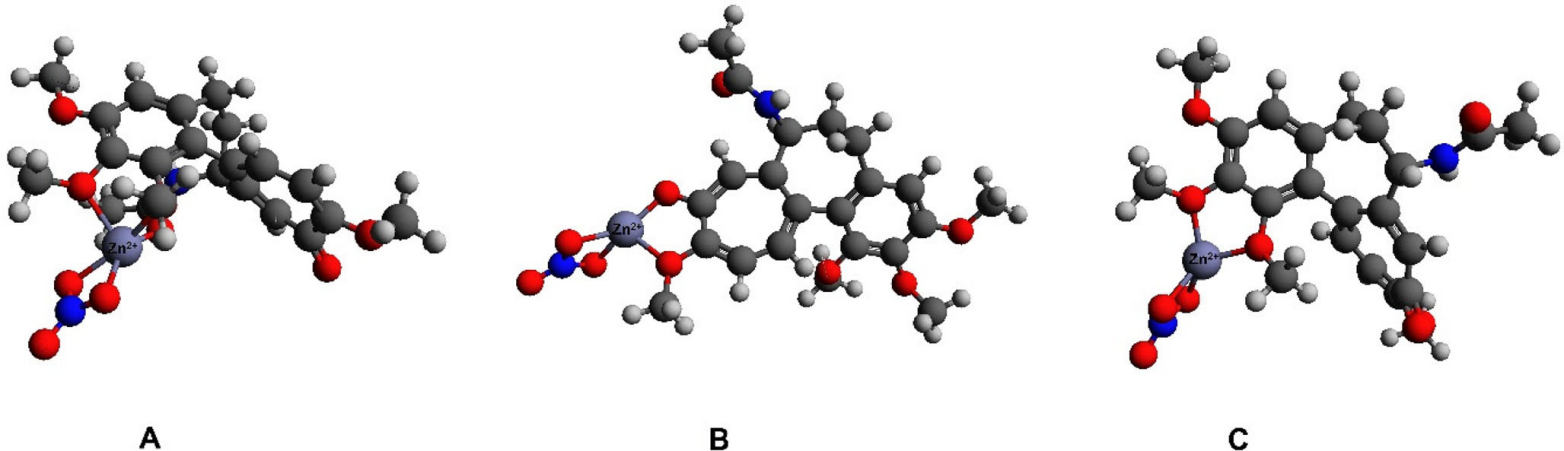
Optimized structures** A**–**C** with 1:1:1 stoichiometry

The initial interaction schemes of the 1:1:1 complex (structures **A**–**C**) consisted of one molecule of colchicine, one zinc cation, and nitrate anion. In structure **A**, colchicine coordinates with the zinc cation via three oxygen atoms (O1, O2, and O4). Structure **B** has the colchicine molecule coordinating to the zinc cation via O5 and O6. The colchicine molecule in structure **C** coordinates via the oxygen atoms O1 and O3. All of these structures have a charge of +1. The optimized 1:1:1 structures are shown in Fig. 4.

**Fig. 5:**
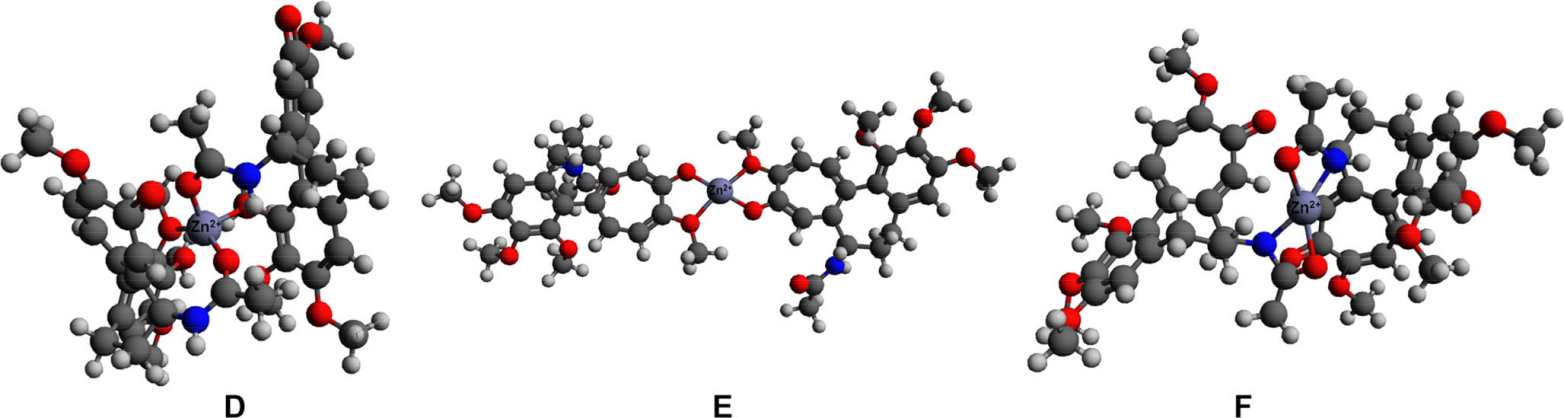
Optimized structures** D**–**F** with 2:1 stoichiometry

Initial interaction schemes of the 2:1 complex of colchicine with Zn(II) (structures **D**–**F**) consisted of two molecules of colchicine and one zinc(II) cation. In structure **D**, both molecules of colchicine are coordinated via O1 and O4, in structure **E** both colchicine molecules are coordinated via O5 and O6, while structure **F** has both colchicine molecules coordinated via O4 and N1. All of these structures have a charge of +2. The optimized 2:1 structures are shown in Fig. 5.

**Fig. 6:**
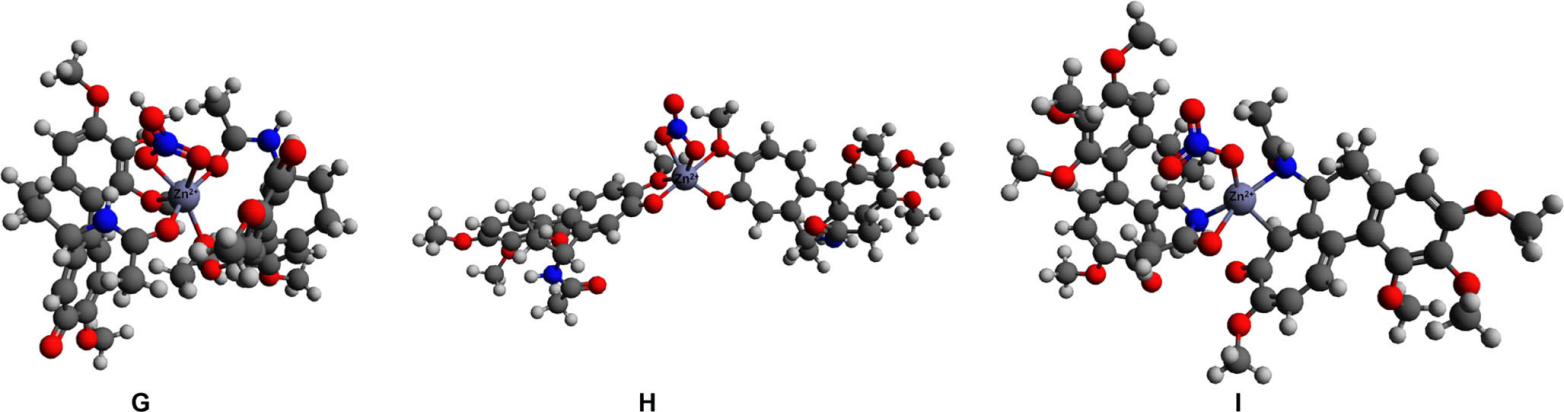
Optimized structures** G**–**I** with 2:1:1 stoichiometry

The initial interaction schemes of the 2:1:1 complex (structures **G**–**I**) consisted of two colchicine molecules, one zinc(II) cation, and one nitrate anion, and all three of these structures have a charge of +1. Structure **G** has both colchicine molecules coordinated to Zn(II) via O1 and O4, structure **H** has both molecules of colchicine coordinated to the zinc cation via O5 and O6, and in structure **I**, O4 and N1 of colchicine coordinate to the central Zn cation. The optimized 2:1:1 structures are shown in Fig. 6.

Table 2 shows the interaction energies for each of the structures** A–I **in vacuum and in the presence of solvent (i.e., methanol, as used in the experimental studies). Table S1 in the ESM presents the extended version of Table 2, including values for the counterpoise energy, BSSE, and the sum of the energies of the monomers.

**Table 2:** Calculated interaction energies for the structures** A**–**I** of complexes of colchicine with zinc(II) nitrate in vacuum and methanol, as generated through the studied interaction schemes

Structure label and stoichiometry	Vacuum		Methanol
	Uncorrected interaction energy ( kcal/mol)	Corrected interaction energy (kcal/mol)	Interaction energy (kcal/mol)
**A** (1:1:1)	−980.3	−970.2	−102.6
**B** (1:1:1)	−965.7	−957.8	−93.0
**C** (1:1:1)	−939.5	−931.5	−79.6
**D** (2:1)	−463.0	−446.8	−105.6
**E** (2:1)	−459.9	−451.6	−101.6
**F** (2:1)	−441.6	−428.1	−87.7
**G** (2:1:1)	−607.4	−583.1	−131.1
**H** (2:1:1)	−601.4	−585.3	−122.4
**I** (2:1:1)	−574.0	−552.8	−103.1

In vacuum, the structure with 1:1:1 stoichiometry that has the most favorable interaction energy (−970.2 kcal/mol) is **A**; **B** was 12.4 kcal/mol less favorable and **C** 38.7 kcal/mol less favorable (see Fig. 3). In methanol, among the 1:1:1 structures, **A** was again the most favorable in terms of interaction energy (−102.6 kcal/mol); **B** and **C** were less favorable by 9.6 kcal/mol and 26 kcal/mol, respectively.

Turning our attention to the 2:1 structures, the most favorable in vacuum was structure **E** (−451.6 kcal/mol), which was more energetically favorable than **D** by 4.8 kcal/mol and **F** by 23.5 kcal/mol. In methanol, the 2:1 structure with the most favorable interaction energy was **D** (−105.6 kcal/mol) instead; **E** and **F** were 4.0 and 17.9 kcal/mol less favorable, respectively.

Among the structures with 2:1:1 stoichiometry, structure** H** (−585.3 kcal/mol) was more energetically favorable than **G** (by 2.2 kcal/mol) and **I** (by 32.5 kcal/mol) in vacuum. In methanol, the most favorable 2:1:1 structure was **G** (−131.1 kcal/mol), with** H** being less favorable by 8.7 kcal/mol and **I** by 28.0 kcal/mol.

Results of the energy calculations for the investigated schemes in vaccuum suggest that, in the presence of one molecule of colchicine, coordination via O1 and O4 is energetically most favorable, but in stoichiometries with two molecules of colchicine, coordination via O5 and O6 is favored. This may be explained by the size of the colchicine molecule, which may cause steric hindrance when coordination is attempted through atoms other than O5 and O6. Calculations show that, in methanol, the structure with the most favorable interaction energy always has one or both molecules of colchicine coordinated via O1 and O4. Our calculations show that colchicine can also coordinate via N1, but this is less favorable in both vacuum and methanol. The atomic coordinates of the obtained structures are included in Tables S2–S4 of the ESM.

Selected interatomic distances, Mulliken point charges, and Wiberg bond indices are shown in Table 3. Table S5 in the ESM includes an extended version of Table 3 that presents rho and its Laplacian for bond critical points between the zinc(II) cation and the coordinating atoms.

**Table 3:** Selected geometric parameters, calculated Mulliken partial charges, and Wiberg bond indices for the structures** A**–**I** of complexes of colchicine with zinc(II) nitrate in vacuum and methanol, as generated through the studied interaction schemes

Structure label and stoichiometry	Mulliken partial charge (in* e*) on the zinc (II) cation	Coordinating atom (CA)	Mulliken partial charge (in* e*) on the CA	Distance between the CA and the cation (Å)	Wiberg bond index for Zn^2+^–CA
**A** (1:1:1)	0.761	O1	−0.478	2.067	0.162
		O2	−0.458	2.190	0.143
		O4	−0.400	1.939	0.278
		O1(NO_3_)	−0.263	2.015	0.291
		O2(NO_3_)	−0.236	2.096	0.244
**B** (1:1:1)	0.788	O5	−0.483	2.100	0.163
		O6	−0.450	1.856	0.398
		O1(NO_3_)	−0.260	2.023	0.299
		O2(NO_3_)	−0.263	2.015	0.306
**C** (1:1:1)	0.836	O1	−0.542	1.964	0.238
		O2	−0.524	1.952	0.247
		O1(NO_3_)	−0.247	1.987	0.336
		O2(NO_3_)	−0.265	2.022	0.299
**D** (2:1)	0.653	O1a	−0.500	2.045	0.120
		O4a	−0.425	1.932	0.191
		O1b	−0.528	1.983	0.128
		O4b	−0.407	1.976	0.182
**E** (2:1)	0.728	O5a	−0.480	2.142	0.155
		O6a	−0.475	1.850	0.398
		O5b	−0.485	2.113	0.158
		O6b	−0.475	1.854	0.400
**F** (2:1)	0.514	N1a	−0.582	2.029	0.247
		O4a	−0.255	2.215	0.175
		N1b	−0.711	2.072	0.232
		O4b	−0.236	2.153	0.197
**G** (2:1:1)	0.694	O1a	−0.486	2.145	0.124
		O4a	−0.362	2.002	0.202
		O1b	−0.473	2.273	0.115
		O4b	−0.415	1.968	0.199
		O1(NO_3_)	−0.329	2.150	0.185
		O2(NO_3_)	−0.234	2.367	0.154
**H** (2:1:1)	0.707	O5a	−0.434	2.213	0.124
		O6a	−0.413	1.959	0.280
		O5b	−0.409	2.466	0.082
		O6b	−0.441	1.925	0.304
		O1(NO_3_)	−0.288	2.152	0.212
		O2(NO_3_)	−0.254	2.104	0.241
**I** (2:1:1)	0.471	N1a	−0.574	2.192	0.141
		N1b	−0.677	2.059	0.208
		O4b	−0.263	2.274	0.161
		O1(NO_3_)	−0.371	1.957	0.283

In all of the structures** A**–**I**, the Zn^2+^…O6 distance is the shortest: 1.856 Å for **B** (1:1:1 stoichiometry); 1.850 Å for **E** (2:1 stoichiometry); 1.925 Å for **H** (2:1:1 stoichiometry). This suggests that the interaction between the central Zn(II) cation and the colchicine ligand is the strongest interaction in the complexes. Calculated Wiberg bond indices also confirmed that Zn^2+^…O6 is the strongest interaction in each complex. The bond index for this bond was highest in each investigated structure.

The Mulliken partial charge on the zinc cation varied with the structure for each stoichiometry. Among the 1:1:1 structures, it ranged from +0.761*e* for **A** to +0.836*e* for **C**. For a stoichiometry of 2:1, it ranged from +0.514*e* for **F** to +0.728*e* for **E**. Among the 2:1:1 structures, it ranged from +0.471*e* for **I** to +0.707*e* for **H**.

The calculated Mulliken point charges for the coordinating O and N atoms also varied with the structure for each complex stoichiometry. For the 1:1:1 structures, they ranged from −0.542*e* for O1 in **C** to −0.400*e* for O4 in **A**. For structures with a stoichiometry of 2:1, they ranged from −0.711*e* for N1b in **F** to −0.236*e* for O4b in **F**. Among the 2:1:1 structures, they ranged from −0.677*e* for N1b in **I** to −0.263*e* for O4b in **I**. As we can see, for both 2:1 and 2:1:1 structures, the calculated Mulliken charges when the colchicine coordinates via an nitrogen atom are most negative for the N1b atom and least negative for the O4b atom.

### NMR measurements

NMR spectra for the colchicine complexes were measured and calculated in CD_3_CN. Selected ^1^H and ^13^C NMR data for colchicine and its complexes with zinc nitrate are given in Tables 4 and 5, respectively (for the full data, see Tables S6–S13 in the ESM). The calculated ^1^H NMR and ^13^C NMR chemical shifts differed from those obtained experimentally. The main reason for those differences is the fact that we do not know which particular complex was examined experimentally. The smallest squared differences between the experimental and calculated chemical shifts were recorded for structure **B** (1:1:1) in the ^1^H NMR spectrum (49.36) and for the uncoordinated colchicine in the ^13^C NMR spectrum (52.50).

**Table 4:** Selected experimental and calculated ^1^H NMR chemical shift data for colchicine and its complexes

Hydrogen atom	Chemical shift (ppm)					
	Experimental		Calculated			
	Colchicine	Colchicine–Zn(NO_3_)_2_	Colchicine	**A** (1:1:1)	**B** (1:1:1)	**C** (1:1:1)
1H on C8	7.25	7.81	7.21	6.99	9.35	7.00
1H on C11	6.93	7.5	7.67	6.79	7.57	6.52
1H on C12	7.16	7.68	7.21	7.24	8.10	6.82
3H on CH_3_O-2	3.86	3.88	2.74	3.97	2.95	2.79
			3.92	5.17	3.94	4.21
			3.62	3.94	3.39	5.49
3H on OCH_3_-10	3.9	4.06	3.28	4.08	4.21	4.06
			3.87	3.85	4.18	3.74
			2.00	1.77	2.07	1.76
1H on NH	7.4	7.55	5.37	5.10	6.19	5.50
∑(calc − exp)^2^			58.14	52.03	49.36	80.90

**Table 5:** Selected experimental and calculated ^13^C NMR chemical shift data for colchicine and its complexes

Carbon atom	Chemical shift (ppm)					
	Experimental		Calculated			
	Colchicine	Colchicine–Zn(NO_3_)_2_	Colchicine	**A** (1:1:1)	**B** (1:1:1)	**C** (1:1:1)
C1a	126.57	125.29	123.46	128.09	121.80	119.28
C3	154.41	157.69	154.70	155.69	156.93	150.12
C4a	136.84	141.92	142.88	142.10	142.51	147.60
C5	30.27	29.71	30.27	29.47	30.85	30.08
C6	36.84	37.15	34.27	38.19	34.67	33.03
C7	52.95	54.22	58.95	65.98	55.40	57.38
C7a	152.01	155.32	147.81	142.22	161.69	146.12
C9	179.63	178.48	178.81	178.10	168.47	183.44
C10	164.88	163.14	163.83	165.01	157.02	163.10
C11	112.98	118.9	118.65	110.50	118.67	108.70
C12	136.66	140.13	141.82	134.16	148.05	138.43
OCH_3_(1)	61.61	61.95	62.63	69.02	62.70	73.42
OCH_3_(10)	56.76	58.33	61.16	59.38	61.94	58.93
C=O(CH_3_)	170.04	172.16	172.36	179.38	175.16	173.14
∑(calc − exp)^2^			52.50	75.08	61.46	79.91

In the ^1^H NMR spectra, a doublet from the amine group (NH) moves from 7.40 ppm for uncoordinated colchinine to 7.55 ppm for its complex with zinc(II) nitrate. This change can also be observed in the calculated data (e.g., from 5.37 for** 1** to 6.19 ppm for **B**). There is also a notable change in the chemical shift calculated for one of the protons on C2 after coordination, from 3.92 in** 1** to 3.94 ppm in **B**. Further, some changes in the chemical shifts of the protons on C10 upon complexation can be observed in both the measured and calculated spectra. Two protons on C11 and C12 that appear as neighboring doublets in the experimental spectrum of colchicine shift markedly after complexation; this phenomenon can also be seen when comparing the calculated spectra for** 1** and **B**. The proton on C8 in the ^1^H NMR spectrum is observed as a singlet that shifts upon complexation. Again, this shift in the singlet from the proton on C8 can be seen by comparing the calculated spectra for** 1** and **B**. In the measured spectra, the singlet due to the proton on C4 does not shift upon complexation: it appears at 6.70 ppm in the spectra for colchicine and its complex with zinc(II) nitrate. Similarly, signals from the protons on C5 and C6 remain almost unchanged after complexation in both the measured and calculated spectra. Finally, the proton signals from the four methoxy groups at C1, C2, C3, and C10 appear as four singlets in the region 3.59–4.06 ppm in the ^1^H NMR spectra measured both before and after complexation (see the ESM).

Switching our attention to the ^13^C NMR spectra, both the experimental and calculated chemical shifts of carbon atoms on ring A (C1a–C4a, see Fig. 1) show some changes after complexation, especially when the spectrum of** 1** is compared to that for complex structure **A** (see Table 5). Some changes are also visible in the experimental chemical shifts for carbon atoms on ring B after complexation: the signal for C5 moves from 30.27 to 29.71 ppm; the signals for C6 and C7 move from 36.84 to 37.15 ppm and from 52.95 to 54.22 ppm, respectively; and the signal for C7a moves from 152.01 to 155.32 ppm. Similar shifts in the signals from these atoms upon complexation are also seen in the calculated spectra: the signal for C5 changes from 30.27 ppm (for **1**) to 29.47 ppm (for structure **A**); the signal for C6 changes from 34.27 (**1**) to 38.19 ppm (**A**); and the signals for C7 and C7a shift from 58.95 (**1**) to 65.98 ppm (**A**) and from 147.81 ppm (**1**) to 161.69 ppm (**B**), respectively. After complexation, the signal from the C4 carbonyl carbon shifts from 179.63 to 178.48 ppm when comparing the measured spectra and from 178.81 to 178.10 ppm when comparing the calculated spectra for** 1** and **A**. Complexation also causes changes in the chemical shifts of the carbon atoms neighboring the oxygen atoms of the methoxy and carbonyl groups: the experimental and calculated signals from C11 and C12 on ring C show marked shifts upon complexation.

It is therefore clear that the experimental and calculated NMR spectral data present similar trends in chemical shift movements upon complexation.

### FT IR measurements

FT IR spectra of the uncoordinated and complexed colchicine were measured in the solid state (i.e., KBr pellets), in nujol, and in CD_3_CN. The corresponding spectra were also calculated in vacuum, a nonpolar solvent (with a dielectric constant of 2.06, a solvent radius of 2.0 Å, a refractive index of 1.4338, and a molar volume of 272 cm^3^/mol), and CD_3_CN. Data for the carbonyl groups are given in Table 6. In the experimental FT IR spectra (in nujol), the band from stretching vibrations of the carbonyl group C13=O4 does not shift much upon complexation, while the band from stretching vibrations of the carbonyl group C9=O6 on tropolone ring C shifts 14 cm^−1^ lower upon complexation. Similar behavior was observed for the latter band in the experimental FT IR spectra obtained with KBr pellets and in CD_3_CN solution; upon complexation, the band shifts from 1680 to 1652 cm^−1^ when using KBr pellets and from 1681 to 1669 cm^−1^ in CD_3_CN solution. Calculated FT IR spectra in the nonpolar solvent show similar results, especially when the spectrum for** 1** is compared to those for complex structures **B** and **C**: the band from carbonyl group C13=O4 does not shift much upon complexation from **1** to **B** or **C**, while the band for carbonyl group C9=O6 shifts towards higher wavenumbers upon complexation from **1** to structure **A** (by 37 cm^−1^) or structure **C** (by 103 cm^−1^). All of the calculated spectra (i.e., those obtained in vacuum, nonpolar solvent, and CD_3_CN) showed similarities. Upon complexation to structure **A**, there are notable changes in the stretching bands for carbonyls C13=O4 and C9=O6, whereas complexation to structure **B** or **C** only significantly changes the band for carbonyl C9=O6 (shifting it towards higher wavenumbers). The full measured and calculated FT IR spectra are given in Figs. S2–S19 of the ESM.

**Table 6:** Experimental and calculated FT IR wavenumbers (*v*) for carbonyl groups of uncomplexed and complexed colchicine (measured in KBr, nujol, or CD_3_CN and calculated in vacuum, nonpolar solvent, or CD_3_CN)

	Measured/calculated in:	Structure or complex	*v*(C13=O4)	*v*(C9=O6)
Experimental data	KBr pellet	**1**	1680	1615
		Colchicine–Zn(NO_3_)_2_	1652	1601
	Nujol	**1**	1656	1614
		Colchicine–Zn(NO_3_)_2_	1652	1600
	CD_3_CN	**1**	1681	1619
		Colchicine–Zn(NO_3_)_2_	1669	1604
Calculated data	Vacuum	**1**	1698	1583
		**A** (1:1:1)	1649	1641
		**B** (1:1:1)	1692	1618
		**C** (1:1:1)	1699	1682
	Nonpolar solvent^a^	**1** (1:1:1)	1697	1581
		**A** (1:1:1)	1646	1657
		**B** (1:1:1)	1697	1618
		**C** (1:1:1)	1700	1684
	CD_3_CN	**1** (1:1:1)	1695	1578
		**A** (1:1:1)	1636	1644
		**B** (1:1:1)	1696	1619
		**C** (1:1:1)	1699	1685

^a^ Parameters: dielectric constant, 2.06; solvent radius, 2.0 Å; refractive index, 1.4338; molar volume, 272 cm^3^/mol

### Bond path and bond critical points

**Fig. 7:**
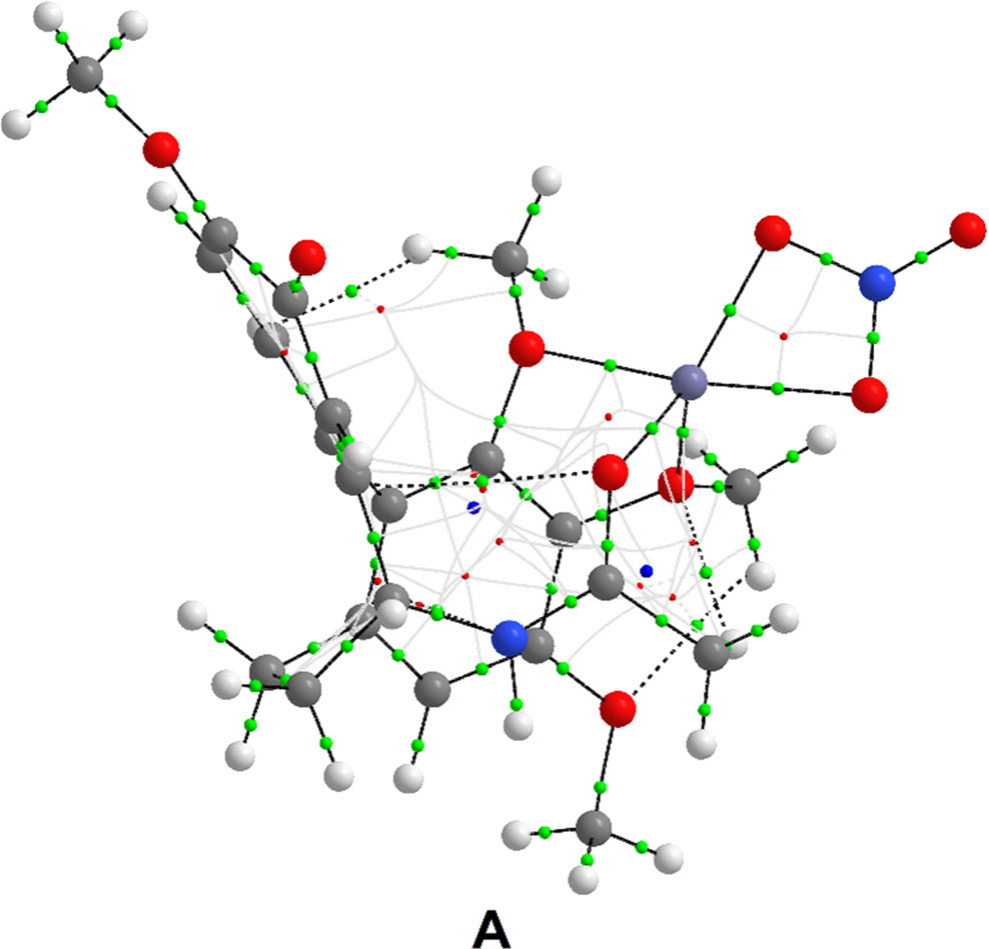
Bond paths (*black*) and bond critical points (*green*) of the most energetically favorable [colchicine + Zn(II) + NO_3_] complex structure** A** (i.e., 1:1:1 stoichiometry)

**Fig. 8:**
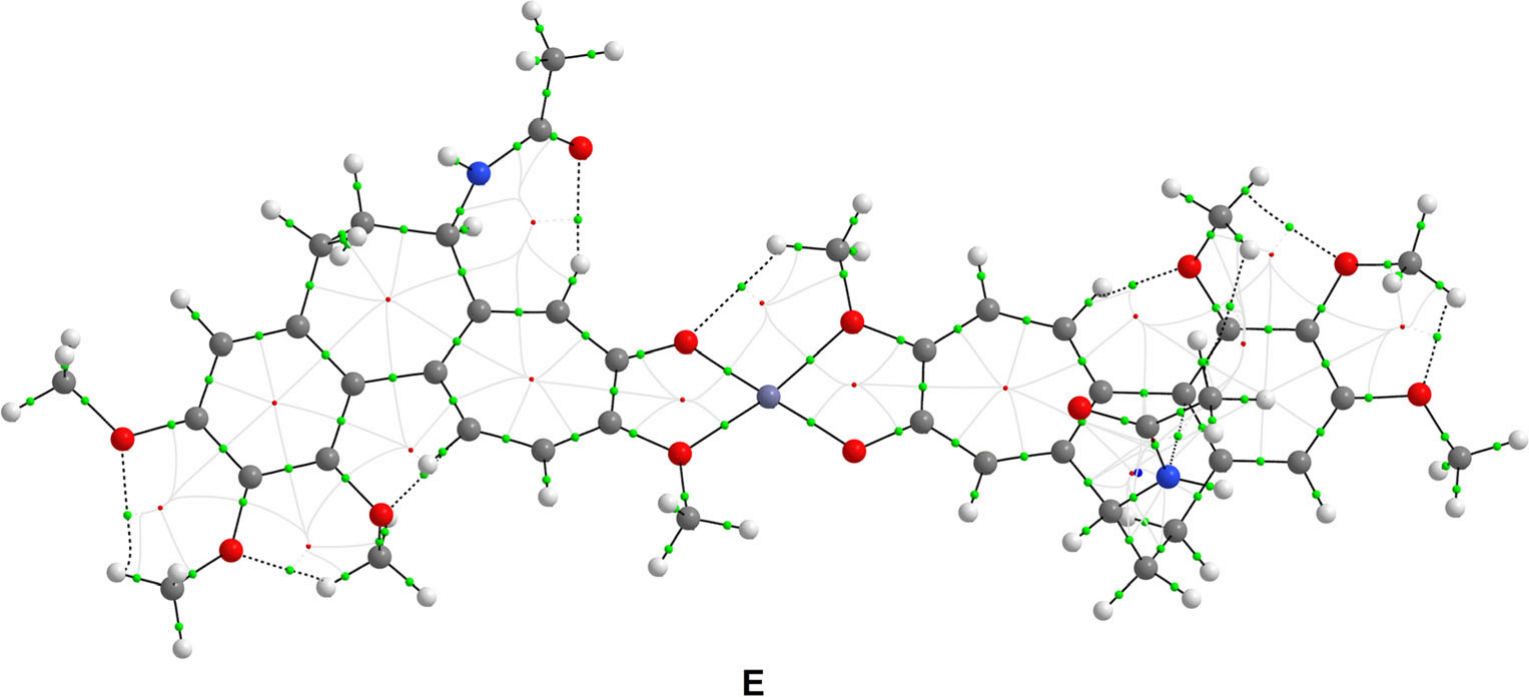
Bond paths (*black*) and bond critical points (*green*) of the most energetically favorable [2 × colchicine + Zn(II)] complex structure** E** (i.e., 2:1 stoichiometry)

**Fig. 9:**
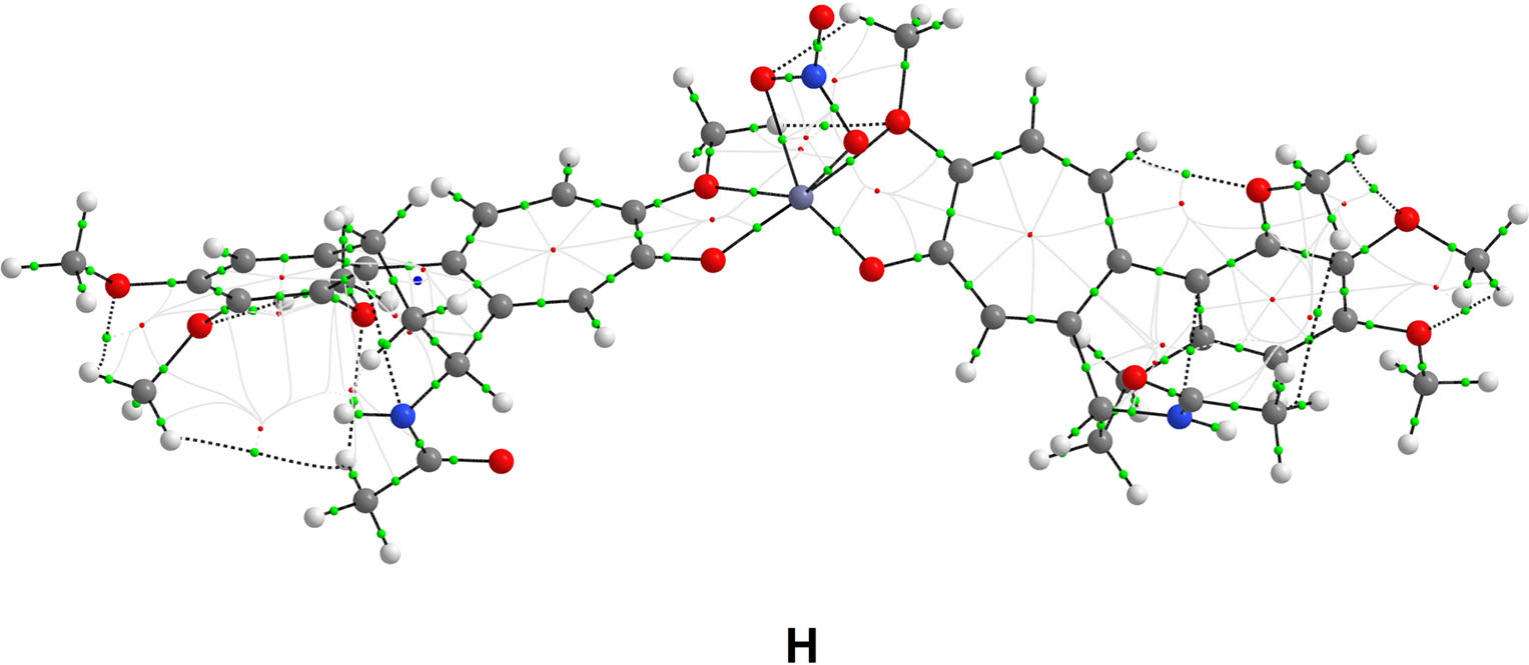
Bond paths (*black*) and bond critical points (*green*) of the most energetically favorable [2 × colchicine + Zn(II) + NO_3_] complex structure** H** (i.e., 2:1:1 stoichiometry)

We generated wfn files for all of the structures of colchicine complexed with Zn(II) and used them to find bond paths and bond critical points using AIMPAC. Figures 7–9 show the resulting complex structures with the lowest interaction energies (other structures are included in Figs. S20–S25 of the ESM). The figures demonstrate that all of the atoms in colchicine that were initially selected as coordinating atoms form bonds with the central zinc cation according to the quantum theory of atoms in molecules. The bond paths and bond critical points indicate that colchicine can coordinate to Zn(II). In one of the investigated complex structures containing a nitrate anion (structure** I** with a stoichiometry of 2:1:1), this anion coordinates to the central zinc cation via one oxygen atom rather than two. The bond paths and bond critical points for this complex (see Fig. S25 in the ESM) indicate that one of the oxygen atoms in the nitrate anion is involved in a hydrogen bond, which may explain why it does not coordinate to the zinc cation.

### Conformation of the seven-membered ring of colchicine

Table 7 presents dihedral angles and calculated amplitudes of the puckering and phase angles for the seven-membered ring in the most energetically favorable structures of each colchicine complex (see Tables S14–S16 in the ESM for the coordinates of the atoms in the seven-membered ring).

**Table 7:** Dihedral angles and calculated puckering amplitudes and phase angles of the seven-membered ring in the most energetically favorable complexes of colchicine with a zinc(II) cation (and, in some cases, a nitrate anion)

		Colchicine complex structure and stoichiometry					Crystal structure	
		**A** (1:1:1)	**E** (2:1)		**H** (2:1:1)		DAECOL^b^	ISCHOL^c^
Dihedral angle (°)	C12a–C1a–C4a–C5	5.4	11.6	−1.6	−5.0	−2.6	−5.6	−6.6
	C1a–C4a–C5–C6	−72.0	60.9	−72.6	−71.1	−71.8	75.6	−68.2
	C4a–C5–C6–C7	31.3	−91.9	46.1	47.0	44.9	−58.9	43.5
	C5–C6–C7–C7a	52.2	48.9	40.4	40.1	41.9	−1.8	44.3
	C6–C7–C7a–C12a	−58.1	−6.7	−76.1	−76.7	−74.9	8.9	−78.1
	C7–C7a–C12a–C1a	−21.2	19.0	7.2	7.4	4.4	45.9	5.6
	C7a–C12a–C1a–C4a	62.3	−47.8	48.1	51.0	51.0	−61.2	52.6
Puckering amplitude (Å)	*ρ*2	1.115	0.686	1.081	1.105	1.093	0.826	1.091
	*ρ*3	0.065	0.408	0.142	0.132	0.130	0.289	0.127
Phase angle (°)	*φ*2	1.1	223.5	16.2	17.3	15.2	183.3	16.7
	*φ*3	63.7	339.5	61.9	60.8	60.2	348.9	53.0
Conformation^a^		TB5	TC1	TB5	TB5	TB5	TC1	TB5

^a^ Notation adapted from Boessenkool and Boyens [37]

^b^ Colchicine-*O*,*N*-diacetate [40]

^c^ Isocolchicine [41]

The calculated parameters of the seven-membered rings in structures **A** (1:1:1) and **H** (2:1:1) suggest that those rings are in a twisted boat conformation (as defined by Cremer and Pople [36]), which is also the case for the seven-membered ring of one of the colchicine molecules in structure **E** (2:1). All of those rings have almost the same puckering amplitudes and phase angles. The calculated parameters for the seven-membered ring of the other colchicine molecule in structure **E** suggest that that ring is in a twisted chair conformation instead. Based on the calculated puckering values for complexes and the crystal structures, we can infer that complexation does not affect the conformation of the seven-membered ring of colchicine.

## Conclusions

In this work, quantum-mechanical computations together with calculated chemical shifts and comparisons with experimental data were used to determine the most probable complexes of colchicine with zinc(II) nitrate in solution. Calculations show that, in methanol, the most probable complex structure with a stoichiometry of 2:1:1 is **G**, while **D** and** A** have the lowest interaction energies of the 2:1 and 1:1:1 complex structures, respectively. In methanol, the most favorable interaction energy is always obtained when one or both molecules of colchicine coordinate to the zinc(II) cation via oxygen atoms O1 and O4. Quantum-mechanical calculations show that, in vacuum, the most probable structure for each complex stoichiometry is **A** (1:1:1), **E** (2:1), and **H** (2:1:1). It was also found that the nitrogen atom of colchicine can act as a donor, but such coordination is significantly less energetically favored than coordination through oxygen atoms.

## Electronic supplementary material

Below is the link to the electronic supplementary material.

ESM 1 (DOCX 5837 kb)

## Abbreviations

DFT: Density functional theory
FT IR: Fourier transform infrared spectroscopy
NMR: Nuclear magnetic resonance
ESI MS: Electrospray ionization mass spectrometry
TRAAK: Potassium channel subfamily K member 4

## References

[1] Cutler S, Cutler H (2000) Biologically active natural products: pharmaceuticals. CRC, New York

[2] Bhat S, Nagasampagi B, Sivakumar M (2005) Chemistry of natural products. Narosa, New Delhi

[3] Capraro H, Brossi A (1984). The alkaloids.

[4] Budavari S (1989). The Merck index: an encyclopedia of chemicals, drugs, and biologicals.

[5] Roubille F, Kritikou E, Busseuil D et al (2013) Colchicine: an old wine in a new bottle? Antiinflamm Antiallergy Agents Med Chem 12:14–23 10.2174/1871523011312010004 23286287

[6] Pelegrín P (2011). Many ways to dilate the P2X7 receptor pore. Br J Pharmacol.

[7] Patel AJ, Honoré E, Lesage F et al (1999) Inhalational anesthetics activate two-pore-domain background K^+^ channels. Nat Neurosci 2:422–426. doi:10.1038/8084 10.1038/8084 10321245

[8] Morrison JD (1951) Preliminary examination of the crystal structures of colchiceine and its copper salt. Acta Crystallogr 4:69–71

[9] Mackay MF, Gable RW, Morrison JD, Satzke LO (1999). Structure of hydrated copper(II) colchiceine. Aust J Chem.

[10] Joanna Kurek WB (2007) ESI MS, spectroscopic and PM5 semiempirical studies of colchicine complexes with lithium, sodium and potassium salts. J Mol Struct 846:13–22. doi:10.1016/j.molstruc.2007.01.004

[11] Ede Bodoki DB (2015) Ab initio study of the Na-colchicine positively charged complex. Farmacia 63:539–542

[12] Parkin G (2007) Applications of tripodal [*S*_3_] and [*Se*_3_] L_2_X donor ligands to zinc, cadmium and mercury chemistry: organometallic and bioinorganic perspectives. New J Chem 31:1996–2014. doi:10.1039/B712012E 10.1039/b712012e PMC2688380 19484137

[13] Malczewska-Jaskóła K, Jankowski W, Warżajtis B et al (2015) Chalcogenated (*S*)-(−)-nicotine derivatives as chiral linkers for 1D coordination polymers. Polyhedron 100:404–411. doi:10.1016/j.poly.2015.08.027

[14] Stanojkovic TP, Kovala-Demertzi D, Primikyri A et al (2010) Zinc(II) complexes of 2-acetyl pyridine 1-(4-fluorophenyl)-piperazinyl thiosemicarbazone: synthesis, spectroscopic study and crystal structures—potential anticancer drugs. J Inorg Biochem 104:467–476. doi:10.1016/j.jinorgbio.2009.12.021 10.1016/j.jinorgbio.2009.12.021 20102782

[15] Andreini C, Banci L, Bertini I, Rosato A (2006). Counting the zinc-proteins encoded in the human genome. J Proteome Res.

[16] Broadley MR, White PJ, Hammond JP (2007). Zinc in plants. New Phytol.

[17] Walkup GK, Burdette SC, Lippard SJ, Tsien RY (2000) A new cell-permeable fluorescent probe for Zn^2+^. J Am Chem Soc 122:5644–5645. doi:10.1021/ja000868p

[18] Hellmich HL, Frederickson CJ, DeWitt DS (2004). Protective effects of zinc chelation in traumatic brain injury correlate with upregulation of neuroprotective genes in rat brain. Neurosci Lett.

[19] Lessinger L, Margulis TN (1978). The crystal structure of colchicine. A new application of magic integers to multiple-solution direct methods. Acta Crystallogr B.

[20] Zhao Y, Truhlar DG (2008). The M06 suite of density functionals for main group thermochemistry, thermochemical kinetics, noncovalent interactions, excited states, and transition elements: two new functionals and systematic testing of four M06-class functionals and 12 other functionals. Theor Chem Accounts.

[21] Dunning TH Jr, Hay PJ (1976) In: Schaefer HF (ed) Modern theoretical chemistry. Plenum, New York, pp 1–28

[22] Bregier-Jarzębowska R, Malczewska-Jaskóła K, Jankowski W (2015). Experimental and quantum-chemical studies of anabasine complexes with copper(II) and zinc(II) ions. Polyhedron.

[23] Mulliken RS (1955). Electronic population analysis on LCAO–MO molecular wave functions. I. J Chem Phys.

[24] Wiberg KB (1968) Application of the Pople–Santry–Segal CNDO method to the cyclopropylcarbinyl and cyclobutyl cation and to bicyclobutane. Tetrahedron 24:1083–1096. doi:10.1016/0040-4020(68)88057-3

[25] Foster JP, Weinhold F (1980). Natural hybrid orbitals. J Am Chem Soc.

[26] Alan E, Reed FW (1983) Natural bond orbital analysis of near-Hartree–Fock water dimer. J Chem Phys 78:4066–4073. doi:10.1063/1.445134

[27] Boys SF, Bernardi F (1970). The calculation of small molecular interactions by the differences of separate total energies. Some procedures with reduced errors. Mol Phys.

[28] Simon S, Duran M, Dannenberg JJ (1996). How does basis set superposition error change the potential surfaces for hydrogen‐bonded dimers?. J Chem Phys.

[29] Jensen F (2008) Basis set convergence of nuclear magnetic shielding constants calculated by density functional methods. J Chem Theory Comput 4:719–727. doi:10.1021/ct800013z 10.1021/ct800013z 26621087

[30] Saielli G, Nicolaou KC, Ortiz A (2011). Addressing the stereochemistry of complex organic molecules by density functional theory-NMR: vannusal B in retrospective. J Am Chem Soc.

[31] Cheeseman JR, Trucks GW, Keith TA, Frisch MJ (1996). A comparison of models for calculating nuclear magnetic resonance shielding tensors. J Chem Phys.

[32] Tomasi J, Mennucci B, Cammi R (2005). Quantum mechanical continuum solvation models. Chem Rev.

[33] Clark T, Chandrasekhar J, Spitznagel GW, Schleyer PVR (1983). Efficient diffuse function-augmented basis sets for anion calculations. III. The 3-21+G basis set for first-row elements, Li–F. J Comput Chem.

[34] Richard F, Bader W (1995). Atoms in molecules: a quantum theory.

[35] Frisch MJ, Trucks GW, Schlegel HB et al (2009) Gaussian 09, revision A.1. Gaussian, Inc., Wallingford

[36] Cremer D, Pople JA (1975). General definition of ring puckering coordinates. J Am Chem Soc.

[37] Boessenkool IK, Boeyens JCA (1980). Identification of the conformational type of seven-membered rings. J Cryst Mol Struct.

[38] Bocian DF, Pickett HM, Rounds TC, Strauss HL (1975). Conformations of cycloheptane. J Am Chem Soc.

[39] Kurek J, Bartkowiak G, Jankowski W (2016). Human body fluid ions in colchicine complexes ESI MS, MADLI MS, spectroscopic, DFT studies and fungicidal activity of colchicine complexes with sodium, potassium, magnesium and calcium carbonates and sulphates. IOSR J Pharm.

[40] Busetta B, Leroy F, Hospital M et al (1979)* O*,*N*-Diacétate de l’énol de colchicine. Acta Crystallogr B 35:1525–1527. doi:10.1107/S056774087900697X

[41] Lessinger L, Margulis TN (1978). The crystal structure of isocolchicine, an inactive isomer of the mitotic spindle inhibitor colchicine. Acta Crystallogr B.

