# Supplementary material for: Quantum-chemical, NMR, FT IR, and ESI MS studies of complexes of colchicine with Zn(II)
Source: J Mol Model. 2017 Mar 20;23(4):127. doi: 10.1007/s00894-017-3306-z (PMC5393104; doi:10.1007/s00894-017-3306-z)
Supplement: Supplementary file 1 — (DOCX 5837 kb) [file 894_2017_3306_MOESM1_ESM.docx]

Supplementary Information

Wojciech Jankowski^1^, Joanna Kurek^1^, Piotr Barczyński^1^, Marcin Hoffmann^1*^

^1^Faculty of Chemistry, Adam Mickiewicz University in Poznan, ul. Umultowska 89, 61-614 Poznań, Poland

****Correspondence to:***

Wojciech Jankowski

Faculty of Chemistry

Adam Mickiewicz University in Poznan

Umultowska 89B,

61-614 Poznań, Poland

Tel: +48 61 829 1554, +48 61 829 1556

E-mail: [wojciech.jankowski89@gmail.com](mailto:wojciech.jankowski89@gmail.com)

**Table S1.** Extended version of Table 2 with calculated energies (counterpoise energy, BSSE, sum of monomers energy, counterpoise uncorrected, corrected interaction energies in vacuum and energy, sum of monomers energy and interaction energy in methanol) for the studied interaction schemes of colchicine with Zn (II) cation.

|  | Vacuum | | | | | Methanol | | |
| --- | --- | --- | --- | --- | --- | --- | --- | --- |
| Colchicine complex | Counterpoise corrected energy [Hartree] | BSSE energy [Hartree] | Sum of monomers [hartree] | Interaction Energy (raw) [kcal/mol] | Interaction Energy (corrected) [kcal/mol] | Energy [Hartree] | Sum of monomers [hartree] | Interaction Energy [kcal/mol] |
| 1:1:1 Type **A** | -1865.502622 | 0.016122 | -1863.956595 | -980.3 | -970.2 | -1865.610714 | -1865.447219 | -102.6 |
| 1:1:1 Type **B** | -1865.493429 | 0.012468 | -1863.967008 | -965.7 | -957.8 | -1865.600880 | -1865.452736 | -93.0 |
| 1:1:1 Type **C** | -1865.455756 | 0.012761 | -1863.971358 | -939.5 | -931.5 | -1865.577779 | -1865.450959 | -79.6 |
| 2:1 Type **D** | -2943.485873 | 0.025829 | -2942.773851 | -463.0 | -446.8 | -2943.710644 | -2943.542387 | -105.6 |
| 2:1 Type **E** | -2943.502461 | 0.013170 | -2942.782727 | -459.9 | -451.6 | -2943.707687 | -2943.545699 | -101.6 |
| 2:1 Type **F** | -2943.388961 | 0.021407 | -2942.706707 | -441.6 | -428.1 | -2943.606790 | -2943.467014 | -87.7 |
| 2:1:1 Type **G** | -3223.899262 | 0.038660 | -3222.969962 | -607.4 | -583.1 | -3224.035552 | -3223.826680 | -131.1 |
| 2:1:1 Type **H** | -3223.935604 | 0.025651 | -3223.002842 | -601.4 | -585.3 | -3224.052099 | -3223.856978 | -122.4 |
| 2:1:1 Type **I** | -3223.813831 | 0.033797 | -3222.932892 | -574.0 | -552.8 | -3223.944526 | -3223.780215 | -103.1 |

Absolute energy baseline [Hartree]: -1865.518744^A^, -1865.505896^B^, -1865.468478^C^,
-2943.511702^D^, -2943.515631^E^, -2943.410369^F^, -3223.937926^G^, -3223.961255^H^, -3223.847609^I^

**Table S2.** Atomic coordinates of optimized 1:1:1 stoichiometry colchicine complexes with zinc cation.

|  | 1:1:1 stoichiometry colchicine complexes atomic coordinates | | | | | | | | |  |
| --- | --- | --- | --- | --- | --- | --- | --- | --- | --- | --- |
|  | Type **A** | | | Type **B** | | | Type **C** | | | |
| Atom | x | y | z | x | y | z | x | y | z | |
| C | 3.994 | -3.849 | 0.793 | -7.813 | -0.012 | 0.335 | -2.262 | 5.295 | -1.310 | |
| O | 3.557 | -2.470 | 0.915 | -6.798 | 0.983 | 0.054 | -2.587 | 3.965 | -0.818 | |
| C | 2.214 | -2.199 | 0.980 | -5.479 | 0.630 | 0.120 | -1.574 | 3.068 | -0.614 | |
| C | 1.181 | -3.142 | 0.821 | -5.017 | -0.638 | 0.505 | -0.205 | 3.331 | -0.790 | |
| C | -0.156 | -2.732 | 0.706 | -3.652 | -0.932 | 0.536 | 0.762 | 2.332 | -0.632 | |
| C | -0.513 | -1.382 | 0.890 | -2.696 | 0.046 | 0.188 | 0.397 | 1.002 | -0.310 | |
| C | 0.510 | -0.477 | 1.176 | -3.176 | 1.329 | -0.224 | -0.962 | 0.784 | -0.037 | |
| O | 0.260 | 0.914 | 1.187 | -2.250 | 2.293 | -0.591 | -1.500 | -0.472 | 0.370 | |
| C | 0.260 | 1.567 | 2.504 | -2.342 | 2.826 | -1.951 | -0.834 | -1.275 | 1.431 | |
| C | 1.862 | -0.843 | 1.122 | -4.547 | 1.631 | -0.245 | -1.935 | 1.787 | -0.177 | |
| O | 2.728 | 0.228 | 0.865 | -4.935 | 2.878 | -0.690 | -3.280 | 1.402 | 0.053 | |
| C | 3.980 | 0.402 | 1.617 | -5.736 | 3.705 | 0.208 | -4.167 | 2.285 | 0.860 | |
| C | -1.855 | -0.864 | 0.519 | -1.224 | -0.134 | 0.310 | 1.350 | -0.151 | -0.361 | |
| C | -2.627 | -0.287 | 1.498 | -0.608 | 0.962 | 0.960 | 0.985 | -1.146 | -1.250 | |
| C | -3.898 | 0.355 | 1.448 | 0.721 | 1.370 | 1.136 | 1.541 | -2.434 | -1.516 | |
| C | -4.625 | 0.780 | 0.356 | 1.873 | 0.817 | 0.611 | 2.306 | -3.207 | -0.669 | |
| O | -5.821 | 1.404 | 0.459 | 3.119 | 1.417 | 0.783 | 2.749 | -4.452 | -0.952 | |
| C | -6.445 | 1.618 | 1.749 | 3.294 | 2.659 | 1.538 | 2.523 | -5.036 | -2.260 | |
| C | -4.225 | 0.679 | -1.081 | 1.976 | -0.394 | -0.144 | 2.679 | -2.804 | 0.722 | |
| O | -4.817 | 1.361 | -1.945 | 3.171 | -0.758 | -0.601 | 2.785 | -3.648 | 1.634 | |
| C | -3.211 | -0.287 | -1.519 | 0.910 | -1.286 | -0.382 | 3.080 | -1.406 | 0.920 | |
| C | -2.193 | -0.953 | -0.888 | -0.477 | -1.263 | -0.172 | 2.545 | -0.224 | 0.474 | |
| C | -1.449 | -1.954 | -1.779 | -1.067 | -2.647 | -0.555 | 3.392 | 1.000 | 0.875 | |
| C | -1.596 | -3.410 | -1.263 | -2.577 | -2.872 | -0.503 | 2.823 | 2.414 | 0.704 | |
| C | -1.238 | -3.663 | 0.228 | -3.219 | -2.347 | 0.795 | 2.220 | 2.682 | -0.688 | |
| N | 0.000 | -1.726 | -2.100 | -0.416 | -3.665 | 0.288 | 4.657 | 0.891 | 0.121 | |
| C | 0.820 | -0.671 | -2.012 | 0.539 | -4.529 | -0.225 | 5.814 | 1.458 | 0.624 | |
| C | 2.227 | -0.828 | -2.526 | 0.943 | -5.675 | 0.665 | 7.069 | 1.237 | -0.183 | |
| O | 0.460 | 0.441 | -1.482 | 1.020 | -4.349 | -1.365 | 5.797 | 2.102 | 1.695 | |
| H | 3.643 | -4.288 | -0.148 | -7.739 | -0.365 | 1.371 | -1.757 | 5.232 | -2.280 | |
| H | 3.641 | -4.443 | 1.644 | -7.731 | -0.856 | -0.360 | -1.638 | 5.834 | -0.588 | |
| H | 5.082 | -3.812 | 0.797 | -8.765 | 0.494 | 0.186 | -3.218 | 5.803 | -1.424 | |
| H | 1.417 | -4.196 | 0.699 | -5.724 | -1.419 | 0.771 | 0.123 | 4.328 | -1.071 | |
| H | -0.532 | 1.126 | 3.115 | -2.195 | 2.015 | -2.675 | -0.273 | -2.082 | 0.958 | |
| H | 0.061 | 2.622 | 2.317 | -1.535 | 3.554 | -2.036 | -1.632 | -1.672 | 2.064 | |
| H | 1.236 | 1.440 | 2.985 | -3.311 | 3.304 | -2.104 | -0.174 | -0.623 | 2.007 | |
| H | 4.398 | 1.348 | 1.272 | -6.722 | 3.260 | 0.359 | -3.621 | 2.599 | 1.753 | |
| H | 4.650 | -0.430 | 1.395 | -5.827 | 4.669 | -0.291 | -5.032 | 1.673 | 1.125 | |
| H | 3.755 | 0.444 | 2.687 | -5.220 | 3.827 | 1.168 | -4.445 | 3.139 | 0.248 | |
| H | -2.219 | -0.372 | 2.507 | -1.312 | 1.642 | 1.430 | 0.143 | -0.897 | -1.899 | |
| H | -4.321 | 0.573 | 2.426 | 0.833 | 2.277 | 1.725 | 1.224 | -2.882 | -2.455 | |
| H | -6.621 | 0.663 | 2.260 | 2.731 | 3.466 | 1.061 | 1.453 | -5.204 | -2.427 | |
| H | -7.394 | 2.101 | 1.528 | 4.362 | 2.873 | 1.498 | 3.048 | -5.989 | -2.242 | |
| H | -5.829 | 2.276 | 2.373 | 2.982 | 2.510 | 2.575 | 2.935 | -4.394 | -3.049 | |
| H | -3.305 | -0.424 | -2.598 | 1.286 | -2.186 | -0.878 | 3.938 | -1.336 | 1.593 | |
| H | -1.946 | -1.923 | -2.757 | -0.718 | -2.867 | -1.574 | 3.666 | 0.909 | 1.936 | |
| H | -0.988 | -4.065 | -1.905 | -2.731 | -3.952 | -0.611 | 3.655 | 3.097 | 0.904 | |
| H | -2.639 | -3.704 | -1.429 | -3.065 | -2.397 | -1.361 | 2.072 | 2.614 | 1.481 | |
| H | -0.939 | -4.710 | 0.347 | -2.510 | -2.408 | 1.636 | 2.756 | 2.090 | -1.443 | |
| H | -2.138 | -3.516 | 0.837 | -4.084 | -2.958 | 1.070 | 2.345 | 3.734 | -0.964 | |
| H | 0.414 | -2.542 | -2.546 | -0.791 | -3.833 | 1.217 | 4.690 | 0.330 | -0.723 | |
| H | 2.931 | -0.774 | -1.684 | 0.558 | -6.607 | 0.240 | 7.552 | 2.201 | -0.365 | |
| H | 2.459 | 0.007 | -3.194 | 0.574 | -5.577 | 1.690 | 6.888 | 0.738 | -1.140 | |
| H | 2.392 | -1.767 | -3.059 | 2.032 | -5.755 | 0.676 | 7.769 | 0.635 | 0.405 | |
| Zn | 1.498 | 1.597 | -0.321 | 4.615 | 0.351 | -0.237 | -3.446 | -0.542 | 0.114 | |
| N | 2.906 | 3.614 | -0.578 | 6.801 | 1.427 | -0.110 | -5.174 | -2.214 | 0.417 | |
| O | 1.825 | 3.509 | 0.222 | 5.896 | 1.675 | -1.073 | -4.589 | -1.910 | -0.765 | |
| O | 3.155 | 2.488 | -1.245 | 6.345 | 0.524 | 0.781 | -6.042 | -3.060 | 0.550 | |
| O | 3.579 | 4.637 | -0.666 | 7.900 | 1.959 | -0.044 | -4.678 | -1.466 | 1.424 | |

**Table S3.** Atomic coordinates of optimized 2:1 stoichiometry colchicine complexes with zinc cation.

|  | 2:1 stoichiometry colchicine complexes atomic coordinates | | | | | | | | |  |
| --- | --- | --- | --- | --- | --- | --- | --- | --- | --- | --- |
|  | Type D | | | Type E | | | Type F | | | |
| Atom | x | y | z | x | y | z | x | y | z | |
| C | -0.055 | -4.147 | -4.500 | 12.707 | 0.092 | -0.120 | -8.609 | -0.411 | 2.920 | |
| O | 0.271 | -3.593 | -3.197 | 11.673 | 1.082 | 0.114 | -7.702 | -1.281 | 2.170 | |
| C | -0.719 | -2.974 | -2.472 | 10.360 | 0.723 | 0.007 | -6.743 | -0.766 | 1.350 | |
| C | -1.965 | -2.583 | -2.993 | 9.919 | -0.561 | -0.348 | -6.429 | -1.522 | 0.204 | |
| C | -2.889 | -1.861 | -2.223 | 8.559 | -0.868 | -0.413 | -5.466 | -1.084 | -0.703 | |
| C | -2.626 | -1.576 | -0.866 | 7.584 | 0.115 | -0.130 | -4.791 | 0.141 | -0.489 | |
| C | -1.352 | -1.921 | -0.393 | 8.044 | 1.417 | 0.249 | -5.042 | 0.858 | 0.704 | |
| O | -0.868 | -1.335 | 0.807 | 7.096 | 2.380 | 0.555 | -4.200 | 1.922 | 0.974 | |
| C | -0.643 | -2.227 | 1.965 | 7.164 | 3.008 | 1.877 | -4.748 | 3.178 | 1.488 | |
| C | -0.389 | -2.596 | -1.154 | 9.412 | 1.735 | 0.297 | -6.002 | 0.405 | 1.633 | |
| O | 0.900 | -2.626 | -0.637 | 9.758 | 3.007 | 0.686 | -6.192 | 1.100 | 2.823 | |
| C | 1.556 | -3.933 | -0.527 | 10.772 | 3.746 | -0.063 | -5.415 | 0.590 | 3.945 | |
| C | -3.676 | -0.954 | -0.002 | 6.120 | -0.081 | -0.284 | -3.831 | 0.699 | -1.485 | |
| C | -4.086 | -1.711 | 1.077 | 5.501 | 0.998 | -0.971 | -4.117 | 1.958 | -2.001 | |
| C | -5.126 | -1.536 | 2.031 | 4.174 | 1.389 | -1.187 | -3.389 | 2.821 | -2.856 | |
| C | -6.054 | -0.522 | 2.178 | 3.013 | 0.844 | -0.667 | -2.043 | 2.797 | -3.195 | |
| O | -7.015 | -0.539 | 3.122 | 1.761 | 1.416 | -0.884 | -1.435 | 3.735 | -3.947 | |
| C | -7.177 | -1.658 | 4.031 | 1.583 | 2.637 | -1.673 | -2.204 | 4.768 | -4.630 | |
| C | -6.156 | 0.735 | 1.374 | 2.917 | -0.337 | 0.126 | -1.083 | 1.802 | -2.690 | |
| O | -6.975 | 1.621 | 1.704 | 1.712 | -0.698 | 0.576 | 0.151 | 2.094 | -2.558 | |
| C | -5.313 | 0.969 | 0.199 | 3.980 | -1.215 | 0.405 | -1.506 | 0.446 | -2.408 | |
| C | -4.270 | 0.306 | -0.396 | 5.368 | -1.207 | 0.199 | -2.670 | -0.059 | -1.849 | |
| C | -3.632 | 1.023 | -1.592 | 5.942 | -2.596 | 0.593 | -2.539 | -1.498 | -1.343 | |
| C | -3.885 | 0.308 | -2.945 | 7.453 | -2.801 | 0.623 | -3.660 | -2.494 | -1.702 | |
| C | -4.096 | -1.223 | -2.858 | 8.149 | -2.293 | -0.654 | -5.071 | -1.910 | -1.896 | |
| N | -2.183 | 1.198 | -1.296 | 5.369 | -3.606 | -0.321 | -2.287 | -1.264 | 0.161 | |
| C | -1.713 | 1.985 | -0.310 | 4.299 | -4.412 | 0.020 | -1.593 | -2.247 | 1.025 | |
| C | -2.490 | 3.166 | 0.167 | 4.038 | -5.578 | -0.899 | -2.062 | -3.640 | 1.178 | |
| O | -0.584 | 1.716 | 0.263 | 3.608 | -4.186 | 1.040 | -0.673 | -1.696 | 1.679 | |
| H | -0.284 | -3.352 | -5.221 | 12.664 | -0.282 | -1.150 | -8.890 | 0.459 | 2.319 | |
| H | -0.901 | -4.840 | -4.426 | 12.615 | -0.738 | 0.590 | -8.152 | -0.075 | 3.854 | |
| H | 0.835 | -4.685 | -4.821 | 13.649 | 0.612 | 0.043 | -9.482 | -1.030 | 3.127 | |
| H | -2.221 | -2.812 | -4.025 | 10.640 | -1.344 | -0.563 | -6.988 | -2.437 | 0.034 | |
| H | -1.258 | -1.850 | 2.786 | 7.049 | 2.242 | 2.653 | -5.734 | 3.370 | 1.050 | |
| H | 0.424 | -2.216 | 2.214 | 6.328 | 3.706 | 1.910 | -4.037 | 3.948 | 1.184 | |
| H | -0.957 | -3.239 | 1.693 | 8.113 | 3.534 | 1.996 | -4.843 | 3.147 | 2.575 | |
| H | 2.536 | -3.739 | -0.090 | 11.773 | 3.444 | 0.247 | -5.662 | -0.461 | 4.143 | |
| H | 1.655 | -4.384 | -1.517 | 10.594 | 4.795 | 0.176 | -5.684 | 1.200 | 4.807 | |
| H | 0.976 | -4.586 | 0.136 | 10.648 | 3.581 | -1.140 | -4.340 | 0.691 | 3.739 | |
| H | -3.552 | -2.655 | 1.201 | 6.209 | 1.668 | -1.449 | -5.091 | 2.350 | -1.709 | |
| H | -5.212 | -2.359 | 2.738 | 4.067 | 2.274 | -1.810 | -3.955 | 3.680 | -3.208 | |
| H | -7.371 | -2.584 | 3.476 | 2.133 | 3.462 | -1.210 | -2.977 | 4.317 | -5.262 | |
| H | -8.042 | -1.403 | 4.639 | 0.511 | 2.833 | -1.647 | -1.480 | 5.296 | -5.247 | |
| H | -6.292 | -1.771 | 4.669 | 1.913 | 2.467 | -2.702 | -2.650 | 5.457 | -3.904 | |
| H | -5.606 | 1.921 | -0.251 | 3.600 | -2.106 | 0.916 | -0.697 | -0.264 | -2.597 | |
| H | -4.064 | 2.026 | -1.671 | 5.535 | -2.836 | 1.584 | -1.600 | -1.938 | -1.690 | |
| H | -3.053 | 0.536 | -3.628 | 7.621 | -3.875 | 0.763 | -3.690 | -3.283 | -0.939 | |
| H | -4.783 | 0.750 | -3.388 | 7.890 | -2.299 | 1.494 | -3.351 | -2.990 | -2.629 | |
| H | -4.260 | -1.612 | -3.869 | 7.480 | -2.372 | -1.526 | -5.774 | -2.735 | -2.047 | |
| H | -5.005 | -1.431 | -2.281 | 9.029 | -2.901 | -0.879 | -5.095 | -1.303 | -2.810 | |
| H | -1.539 | 0.498 | -1.674 | 5.895 | -3.831 | -1.160 | -3.192 | -0.978 | 0.587 | |
| H | -2.954 | 3.700 | -0.665 | 4.439 | -6.489 | -0.442 | -3.149 | -3.675 | 1.319 | |
| H | -1.804 | 3.846 | 0.676 | 4.493 | -5.458 | -1.887 | -1.557 | -4.112 | 2.021 | |
| H | -3.272 | 2.855 | 0.872 | 2.961 | -5.718 | -1.009 | -1.805 | -4.195 | 0.266 | |
| Zn | 0.461 | 0.093 | 0.192 | 0.227 | 0.307 | 0.119 | -0.848 | 0.137 | 0.448 | |
| C | 6.694 | -3.271 | 2.015 | -11.965 | 1.208 | 1.948 | 7.839 | 2.482 | 3.612 | |
| O | 5.323 | -2.899 | 2.320 | -11.269 | 0.143 | 1.252 | 7.917 | 1.994 | 2.252 | |
| C | 4.828 | -1.713 | 1.845 | -9.919 | 0.229 | 1.062 | 6.870 | 1.276 | 1.733 | |
| C | 5.519 | -0.863 | 0.961 | -9.140 | 1.339 | 1.433 | 5.665 | 1.038 | 2.417 | |
| C | 4.945 | 0.321 | 0.482 | -7.754 | 1.326 | 1.266 | 4.666 | 0.250 | 1.840 | |
| C | 3.667 | 0.695 | 0.935 | -7.115 | 0.196 | 0.709 | 4.866 | -0.333 | 0.572 | |
| C | 2.952 | -0.214 | 1.723 | -7.907 | -0.915 | 0.305 | 6.070 | -0.074 | -0.130 | |
| O | 1.554 | 0.006 | 1.845 | -7.231 | -1.976 | -0.252 | 6.156 | -0.609 | -1.394 | |
| C | 1.038 | 0.487 | 3.136 | -7.810 | -2.766 | -1.332 | 7.417 | -1.124 | -1.922 | |
| C | 3.494 | -1.403 | 2.225 | -9.304 | -0.909 | 0.482 | 7.060 | 0.763 | 0.426 | |
| O | 2.659 | -2.229 | 2.944 | -9.996 | -2.069 | 0.212 | 8.195 | 0.982 | -0.319 | |
| C | 3.169 | -2.922 | 4.134 | -11.155 | -2.010 | -0.671 | 8.777 | 2.317 | -0.398 | |
| C | 2.985 | 1.926 | 0.442 | -5.649 | 0.176 | 0.522 | 3.843 | -1.255 | 0.011 | |
| C | 2.769 | 2.962 | 1.330 | -4.983 | -0.977 | 0.967 | 4.257 | -2.532 | -0.367 | |
| C | 2.103 | 4.208 | 1.165 | -3.624 | -1.316 | 1.004 | 3.560 | -3.674 | -0.819 | |
| C | 1.272 | 4.620 | 0.139 | -2.517 | -0.567 | 0.640 | 2.202 | -3.918 | -1.002 | |
| O | 0.608 | 5.796 | 0.140 | -1.228 | -1.074 | 0.783 | 1.709 | -5.119 | -1.374 | |
| C | 0.854 | 6.796 | 1.168 | -0.948 | -2.409 | 1.325 | 2.586 | -6.245 | -1.654 | |
| C | 0.925 | 3.827 | -1.072 | -2.492 | 0.754 | 0.088 | 1.135 | -2.931 | -0.833 | |
| O | -0.155 | 4.017 | -1.684 | -1.305 | 1.303 | -0.195 | -0.102 | -3.254 | -0.939 | |
| C | 1.876 | 2.859 | -1.610 | -3.632 | 1.515 | -0.233 | 1.421 | -1.550 | -0.567 | |
| C | 2.669 | 1.950 | -0.962 | -5.002 | 1.297 | -0.097 | 2.488 | -0.810 | -0.105 | |
| C | 3.244 | 0.753 | -1.731 | -5.871 | 2.410 | -0.700 | 2.201 | 0.614 | 0.382 | |
| C | 4.759 | 0.873 | -1.979 | -6.464 | 3.318 | 0.399 | 2.167 | 0.665 | 1.926 | |
| C | 5.543 | 1.123 | -0.652 | -6.942 | 2.540 | 1.643 | 3.378 | -0.030 | 2.579 | |
| N | 2.485 | 0.481 | -2.955 | -6.915 | 1.876 | -1.572 | 0.877 | 1.125 | -0.139 | |
| C | 1.189 | 0.140 | -2.894 | -6.579 | 1.084 | -2.646 | 0.370 | 2.365 | 0.443 | |
| C | 0.420 | 0.003 | -4.170 | -7.717 | 0.660 | -3.538 | 1.099 | 3.641 | 0.293 | |
| O | 0.571 | 0.011 | -1.779 | -5.383 | 0.773 | -2.857 | -0.757 | 2.221 | 0.981 | |
| H | 7.393 | -2.511 | 2.384 | -11.908 | 2.147 | 1.384 | 7.693 | 1.654 | 4.316 | |
| H | 6.827 | -3.420 | 0.937 | -11.557 | 1.343 | 2.956 | 7.034 | 3.219 | 3.722 | |
| H | 6.863 | -4.211 | 2.537 | -13.002 | 0.882 | 2.013 | 8.799 | 2.960 | 3.805 | |
| H | 6.510 | -1.145 | 0.613 | -9.606 | 2.208 | 1.889 | 5.514 | 1.435 | 3.417 | |
| H | 1.274 | -0.258 | 3.900 | -8.353 | -2.116 | -2.032 | 8.012 | -0.320 | -2.356 | |
| H | 1.496 | 1.454 | 3.365 | -8.486 | -3.524 | -0.935 | 7.128 | -1.846 | -2.688 | |
| H | -0.042 | 0.601 | 3.015 | -6.959 | -3.223 | -1.837 | 7.997 | -1.613 | -1.132 | |
| H | 3.752 | -3.796 | 3.845 | -11.961 | -1.436 | -0.209 | 7.989 | 3.071 | -0.521 | |
| H | 2.282 | -3.213 | 4.697 | -11.460 | -3.046 | -0.820 | 9.416 | 2.299 | -1.281 | |
| H | 3.790 | -2.243 | 4.729 | -10.873 | -1.560 | -1.635 | 9.361 | 2.535 | 0.497 | |
| H | 3.207 | 2.818 | 2.319 | -5.638 | -1.747 | 1.363 | 5.328 | -2.694 | -0.265 | |
| H | 2.204 | 4.887 | 2.009 | -3.423 | -2.306 | 1.408 | 4.212 | -4.524 | -1.008 | |
| H | 0.529 | 6.430 | 2.148 | 0.139 | -2.494 | 1.310 | 3.267 | -6.006 | -2.479 | |
| H | 0.255 | 7.656 | 0.876 | -1.403 | -3.170 | 0.685 | 1.922 | -7.055 | -1.946 | |
| H | 1.915 | 7.070 | 1.192 | -1.321 | -2.476 | 2.351 | 3.151 | -6.528 | -0.759 | |
| H | 1.732 | 2.747 | -2.685 | -3.349 | 2.440 | -0.735 | 0.497 | -1.004 | -0.732 | |
| H | 3.099 | -0.139 | -1.103 | -5.211 | 3.019 | -1.331 | 2.999 | 1.275 | 0.012 | |
| H | 4.966 | 1.698 | -2.674 | -7.289 | 3.894 | -0.040 | 1.243 | 0.180 | 2.282 | |
| H | 5.107 | -0.059 | -2.443 | -5.701 | 4.042 | 0.705 | 2.137 | 1.718 | 2.245 | |
| H | 5.511 | 2.193 | -0.414 | -7.532 | 3.209 | 2.276 | 3.192 | -1.112 | 2.605 | |
| H | 6.593 | 0.862 | -0.813 | -6.065 | 2.239 | 2.232 | 3.455 | 0.299 | 3.620 | |
| H | 2.935 | 0.571 | -3.861 | -7.888 | 2.081 | -1.371 | 0.892 | 1.270 | -1.191 | |
| H | -0.263 | -0.850 | -4.098 | -7.807 | 1.360 | -4.375 | 0.645 | 4.426 | 0.897 | |
| H | 1.065 | -0.115 | -5.044 | -8.678 | 0.633 | -3.011 | 1.061 | 3.927 | -0.769 | |
| H | -0.187 | 0.908 | -4.306 | -7.497 | -0.324 | -3.958 | 2.159 | 3.528 | 0.547 | |

**Table S4.** Atomic coordinates of optimized 2:1:1 stoichiometry colchicine complexes with zinc cation.

|  | 2:1:1 stoichiometry colchicine complexes atomic coordinates | | | | | | | | |
| --- | --- | --- | --- | --- | --- | --- | --- | --- | --- |
|  | Type G | | | Type H | | | Type I | | |
| Atom | x | y | z | x | y | z | x | y | z |
| C | 3.541 | 4.810 | 3.787 | -12.312 | 0.455 | 0.078 | 10.504 | -1.609 | -0.007 |
| O | 2.724 | 3.616 | 3.736 | -11.407 | -0.518 | -0.494 | 9.169 | -2.178 | 0.098 |
| C | 2.973 | 2.689 | 2.755 | -10.053 | -0.327 | -0.351 | 8.027 | -1.432 | 0.071 |
| C | 4.031 | 2.766 | 1.840 | -9.478 | 0.717 | 0.392 | 6.852 | -2.163 | -0.189 |
| C | 4.220 | 1.781 | 0.863 | -8.089 | 0.868 | 0.463 | 5.599 | -1.561 | -0.216 |
| C | 3.344 | 0.678 | 0.780 | -7.246 | -0.008 | -0.251 | 5.456 | -0.172 | 0.021 |
| C | 2.221 | 0.681 | 1.635 | -7.824 | -1.081 | -0.969 | 6.645 | 0.565 | 0.246 |
| O | 1.152 | -0.190 | 1.332 | -6.960 | -2.024 | -1.500 | 6.571 | 1.941 | 0.460 |
| C | 0.716 | -1.152 | 2.352 | -7.113 | -2.413 | -2.896 | 7.140 | 2.771 | -0.601 |
| C | 2.040 | 1.634 | 2.645 | -9.219 | -1.273 | -0.992 | 7.917 | -0.044 | 0.292 |
| O | 0.874 | 1.591 | 3.389 | -9.708 | -2.366 | -1.681 | 9.047 | 0.738 | 0.523 |
| C | 1.021 | 1.511 | 4.839 | -10.667 | -3.236 | -1.015 | 9.212 | 1.185 | 1.901 |
| C | 3.655 | -0.477 | -0.115 | -5.766 | 0.148 | -0.182 | 4.150 | 0.548 | 0.122 |
| C | 3.663 | -1.721 | 0.485 | -5.090 | 0.208 | -1.393 | 4.092 | 1.417 | 1.232 |
| C | 3.964 | -3.023 | -0.007 | -3.722 | 0.386 | -1.695 | 3.211 | 2.422 | 1.669 |
| C | 4.479 | -3.434 | -1.220 | -2.662 | 0.660 | -0.857 | 2.076 | 2.955 | 1.077 |
| O | 4.727 | -4.735 | -1.509 | -1.373 | 0.808 | -1.338 | 1.374 | 3.969 | 1.608 |
| C | 4.450 | -5.782 | -0.552 | -1.081 | 0.804 | -2.757 | 1.801 | 4.639 | 2.826 |
| C | 4.867 | -2.572 | -2.374 | -2.690 | 0.853 | 0.582 | 1.446 | 2.457 | -0.158 |
| O | 5.450 | -3.073 | -3.363 | -1.606 | 1.241 | 1.178 | 0.376 | 3.003 | -0.575 |
| C | 4.510 | -1.153 | -2.407 | -3.828 | 0.575 | 1.391 | 1.930 | 1.283 | -0.851 |
| C | 3.999 | -0.253 | -1.505 | -5.140 | 0.238 | 1.110 | 3.058 | 0.433 | -0.788 |
| C | 3.711 | 1.146 | -2.068 | -6.010 | -0.006 | 2.351 | 2.906 | -0.664 | -1.869 |
| C | 4.680 | 2.238 | -1.556 | -6.892 | 1.228 | 2.650 | 3.975 | -1.742 | -2.032 |
| C | 5.300 | 1.948 | -0.174 | -7.487 | 1.896 | 1.388 | 4.416 | -2.358 | -0.689 |
| N | 2.295 | 1.458 | -1.776 | -6.818 | -1.217 | 2.219 | 1.598 | -1.382 | -1.621 |
| C | 1.271 | 0.796 | -2.353 | -6.228 | -2.425 | 1.911 | 0.775 | -1.623 | -2.788 |
| C | 1.443 | 0.175 | -3.710 | -7.181 | -3.552 | 1.603 | 0.015 | -2.919 | -2.772 |
| O | 0.143 | 0.665 | -1.769 | -4.983 | -2.535 | 1.844 | 0.742 | -0.789 | -3.697 |
| H | 3.469 | 5.370 | 2.847 | -12.230 | 0.477 | 1.172 | 10.559 | -0.879 | -0.819 |
| H | 4.588 | 4.561 | 4.000 | -12.117 | 1.455 | -0.331 | 10.806 | -1.135 | 0.931 |
| H | 3.134 | 5.409 | 4.601 | -13.311 | 0.130 | -0.209 | 11.148 | -2.463 | -0.217 |
| H | 4.721 | 3.605 | 1.873 | -10.108 | 1.427 | 0.922 | 6.964 | -3.226 | -0.379 |
| H | 0.461 | -2.068 | 1.810 | -7.094 | -1.523 | -3.541 | 6.599 | 2.594 | -1.539 |
| H | -0.154 | -0.745 | 2.874 | -6.256 | -3.051 | -3.113 | 7.004 | 3.803 | -0.280 |
| H | 1.536 | -1.325 | 3.058 | -8.051 | -2.950 | -3.049 | 8.205 | 2.548 | -0.728 |
| H | 0.009 | 1.405 | 5.232 | -11.646 | -2.756 | -0.956 | 9.269 | 0.321 | 2.576 |
| H | 1.490 | 2.419 | 5.226 | -10.716 | -4.137 | -1.628 | 10.150 | 1.741 | 1.928 |
| H | 1.620 | 0.632 | 5.112 | -10.314 | -3.491 | -0.007 | 8.381 | 1.837 | 2.194 |
| H | 3.393 | -1.716 | 1.541 | -5.721 | 0.105 | -2.273 | 4.914 | 1.272 | 1.928 |
| H | 3.796 | -3.808 | 0.728 | -3.492 | 0.329 | -2.757 | 3.499 | 2.862 | 2.621 |
| H | 5.065 | -5.661 | 0.348 | -1.598 | 1.635 | -3.250 | 2.800 | 5.069 | 2.698 |
| H | 4.717 | -6.707 | -1.059 | 0.000 | 0.944 | -2.820 | 1.072 | 5.431 | 2.979 |
| H | 3.385 | -5.799 | -0.283 | -1.358 | -0.156 | -3.204 | 1.786 | 3.943 | 3.672 |
| H | 4.724 | -0.766 | -3.406 | -3.560 | 0.626 | 2.447 | 1.350 | 1.175 | -1.779 |
| H | 3.816 | 1.101 | -3.158 | -5.326 | -0.158 | 3.196 | 2.768 | -0.151 | -2.830 |
| H | 4.146 | 3.197 | -1.535 | -7.695 | 0.920 | 3.335 | 3.563 | -2.504 | -2.709 |
| H | 5.492 | 2.339 | -2.285 | -6.283 | 1.967 | 3.185 | 4.852 | -1.328 | -2.542 |
| H | 5.958 | 2.782 | 0.094 | -8.245 | 2.623 | 1.696 | 3.595 | -2.337 | 0.048 |
| H | 5.933 | 1.054 | -0.237 | -6.696 | 2.460 | 0.877 | 4.697 | -3.406 | -0.819 |
| H | 2.070 | 1.965 | -0.909 | -7.830 | -1.144 | 2.230 | 1.718 | -2.239 | -1.062 |
| H | 1.970 | 0.837 | -4.402 | -6.708 | -4.505 | 1.844 | 0.693 | -3.732 | -3.066 |
| H | 0.451 | -0.057 | -4.105 | -8.127 | -3.462 | 2.147 | -0.372 | -3.161 | -1.774 |
| H | 2.010 | -0.762 | -3.637 | -7.388 | -3.534 | 0.522 | -0.797 | -2.876 | -3.501 |
| Zn | -0.412 | 0.688 | 0.155 | -0.022 | 1.747 | 0.143 | 0.395 | -0.313 | -0.134 |
| C | -4.174 | -6.044 | 2.080 | 11.263 | -3.605 | -1.284 | -4.091 | -5.782 | -0.463 |
| O | -2.977 | -5.319 | 1.717 | 10.986 | -2.230 | -0.932 | -4.795 | -4.904 | 0.440 |
| C | -3.108 | -4.082 | 1.122 | 9.678 | -1.826 | -0.805 | -4.825 | -3.558 | 0.158 |
| C | -4.340 | -3.441 | 0.912 | 8.577 | -2.690 | -0.893 | -4.280 | -2.986 | -1.003 |
| C | -4.415 | -2.229 | 0.220 | 7.271 | -2.189 | -0.822 | -4.372 | -1.608 | -1.227 |
| C | -3.236 | -1.667 | -0.330 | 7.049 | -0.810 | -0.643 | -5.021 | -0.773 | -0.293 |
| C | -1.997 | -2.232 | 0.014 | 8.163 | 0.059 | -0.520 | -5.559 | -1.351 | 0.880 |
| O | -0.825 | -1.506 | -0.277 | 7.875 | 1.393 | -0.341 | -6.074 | -0.492 | 1.835 |
| C | 0.149 | -2.178 | -1.147 | 8.704 | 2.244 | 0.497 | -7.420 | -0.749 | 2.332 |
| C | -1.909 | -3.434 | 0.747 | 9.480 | -0.437 | -0.609 | -5.439 | -2.734 | 1.128 |
| O | -0.644 | -3.936 | 1.006 | 10.521 | 0.470 | -0.655 | -5.940 | -3.225 | 2.316 |
| C | -0.367 | -4.594 | 2.276 | 11.613 | 0.326 | 0.295 | -5.068 | -4.052 | 3.144 |
| C | -3.310 | -0.392 | -1.097 | 5.665 | -0.275 | -0.546 | -5.014 | 0.710 | -0.481 |
| C | -2.837 | -0.349 | -2.388 | 5.357 | 0.795 | -1.383 | -6.223 | 1.371 | -0.488 |
| C | -2.825 | 0.713 | -3.335 | 4.154 | 1.493 | -1.612 | -6.551 | 2.743 | -0.666 |
| C | -3.362 | 1.983 | -3.273 | 2.894 | 1.302 | -1.076 | -5.754 | 3.835 | -0.944 |
| O | -3.239 | 2.874 | -4.290 | 1.805 | 2.045 | -1.487 | -6.262 | 5.084 | -1.105 |
| C | -2.484 | 2.554 | -5.476 | 1.975 | 3.187 | -2.376 | -7.682 | 5.334 | -1.001 |
| C | -4.183 | 2.583 | -2.178 | 2.511 | 0.352 | -0.052 | -4.273 | 3.867 | -1.121 |
| O | -4.764 | 3.678 | -2.362 | 1.279 | 0.345 | 0.365 | -3.703 | 4.944 | -1.422 |
| C | -4.292 | 1.932 | -0.877 | 3.404 | -0.575 | 0.551 | -3.456 | 2.670 | -0.926 |
| C | -3.926 | 0.706 | -0.399 | 4.747 | -0.875 | 0.379 | -3.729 | 1.354 | -0.647 |
| C | -4.239 | 0.389 | 1.063 | 5.264 | -1.984 | 1.308 | -2.563 | 0.375 | -0.547 |
| C | -5.640 | -0.212 | 1.182 | 5.458 | -3.308 | 0.540 | -2.337 | -0.334 | -1.890 |
| C | -5.708 | -1.433 | 0.199 | 6.095 | -3.133 | -0.857 | -3.648 | -0.979 | -2.394 |
| N | -3.869 | 1.507 | 1.935 | 6.488 | -1.601 | 2.011 | -1.258 | 0.913 | -0.067 |
| C | -2.543 | 1.721 | 2.096 | 6.485 | -0.533 | 2.880 | -1.102 | 1.120 | 1.375 |
| C | -2.082 | 2.948 | 2.815 | 7.798 | -0.259 | 3.574 | -2.216 | 1.625 | 2.207 |
| O | -1.721 | 0.868 | 1.613 | 5.444 | 0.130 | 3.082 | 0.036 | 0.825 | 1.802 |
| H | -4.813 | -6.205 | 1.203 | 10.926 | -4.291 | -0.497 | -4.555 | -5.776 | -1.458 |
| H | -4.736 | -5.516 | 2.860 | 10.790 | -3.867 | -2.238 | -3.033 | -5.497 | -0.539 |
| H | -3.831 | -7.004 | 2.464 | 12.346 | -3.669 | -1.383 | -4.173 | -6.778 | -0.029 |
| H | -5.256 | -3.884 | 1.292 | 8.722 | -3.756 | -1.047 | -3.794 | -3.611 | -1.749 |
| H | 0.218 | -3.231 | -0.863 | 9.026 | 1.702 | 1.397 | -7.474 | -1.730 | 2.810 |
| H | -0.161 | -2.061 | -2.192 | 9.578 | 2.598 | -0.052 | -7.622 | 0.045 | 3.051 |
| H | 1.112 | -1.690 | -0.972 | 8.060 | 3.077 | 0.784 | -8.141 | -0.697 | 1.505 |
| H | -0.803 | -4.024 | 3.107 | 12.160 | -0.603 | 0.117 | -4.040 | -3.669 | 3.116 |
| H | 0.722 | -4.606 | 2.360 | 12.262 | 1.187 | 0.127 | -5.471 | -3.976 | 4.155 |
| H | -0.769 | -5.607 | 2.283 | 11.229 | 0.347 | 1.326 | -5.085 | -5.087 | 2.798 |
| H | -2.398 | -1.279 | -2.753 | 6.190 | 1.153 | -1.982 | -7.089 | 0.730 | -0.329 |
| H | -2.319 | 0.454 | -4.264 | 4.246 | 2.286 | -2.350 | -7.618 | 2.942 | -0.589 |
| H | -1.442 | 2.311 | -5.225 | 0.976 | 3.609 | -2.487 | -8.054 | 5.081 | 0.000 |
| H | -2.516 | 3.455 | -6.087 | 2.643 | 3.926 | -1.917 | -7.795 | 6.402 | -1.177 |
| H | -2.943 | 1.720 | -6.021 | 2.363 | 2.863 | -3.348 | -8.236 | 4.770 | -1.762 |
| H | -4.782 | 2.614 | -0.177 | 2.890 | -1.124 | 1.340 | -2.408 | 2.962 | -1.049 |
| H | -3.558 | -0.404 | 1.380 | 4.497 | -2.138 | 2.077 | -2.820 | -0.393 | 0.188 |
| H | -6.420 | 0.510 | 0.908 | 6.070 | -3.976 | 1.162 | -1.931 | 0.369 | -2.634 |
| H | -5.817 | -0.546 | 2.213 | 4.479 | -3.789 | 0.424 | -1.595 | -1.134 | -1.721 |
| H | -5.909 | -1.055 | -0.811 | 6.407 | -4.114 | -1.231 | -4.268 | -0.213 | -2.874 |
| H | -6.553 | -2.069 | 0.481 | 5.331 | -2.757 | -1.551 | -3.413 | -1.727 | -3.160 |
| H | -4.545 | 2.166 | 2.305 | 7.355 | -2.084 | 1.803 | -0.931 | 1.783 | -0.561 |
| H | -2.074 | 3.786 | 2.105 | 7.850 | -0.834 | 4.505 | -1.921 | 1.625 | 3.256 |
| H | -1.058 | 2.794 | 3.168 | 8.657 | -0.535 | 2.949 | -2.488 | 2.639 | 1.888 |
| H | -2.744 | 3.215 | 3.644 | 7.851 | 0.800 | 3.832 | -3.112 | 1.002 | 2.080 |
| N | -0.262 | 3.339 | -0.186 | -0.002 | 4.285 | 0.033 | -1.016 | -2.562 | 1.095 |
| O | 0.678 | 2.533 | 0.335 | -0.703 | 3.546 | -0.824 | 0.133 | -2.155 | 0.470 |
| O | -0.042 | 4.555 | -0.355 | 0.667 | 3.574 | 0.927 | -1.232 | -3.792 | 1.143 |
| O | -1.388 | 2.751 | -0.472 | 0.035 | 5.525 | -0.019 | -1.783 | -1.659 | 1.559 |

**Table S5.** Selected geometry parameters, calculated Mulliken partial charges, Wiberg bond indices and bond critical points Rho and its Laplacian for each interaction schemes of colchicine complexes.

| Colchicine complex | Zinc cation Mulliken partial charge | Coordinating Atom (CA) | Coordinating atom Mulliken partial charge | Distance between coordinating atom and cation [Å] | Wiberg Bond Indices  (Zn^2+^-CA) | BCP ρ[a.u.] | BCP ∇^2^[a.u.] |
| --- | --- | --- | --- | --- | --- | --- | --- |
| 1:1:1 Type **A** | 0.761 | O1 | -0.478 | 2.067 | 0.1624 | 0.068 | 0.274 |
|  |  | O2 | -0.458 | 2.190 | 0.1434 | 0.049 | 0.212 |
|  |  | O4 | -0.400 | 1.939 | 0.2782 | 0.090 | 0.373 |
|  |  | O1(nitr) | -0.263 | 2.015 | 0.2905 | 0.081 | 0.303 |
|  |  | O2(nitr) | -0.236 | 2.096 | 0.2436 | 0.067 | 0.252 |
| 1:1:1 Type **B** | 0.788 | O5 | -0.483 | 2.100 | 0.1625 | 0.061 | 0.261 |
|  |  | O6 | -0.450 | 1.856 | 0.3975 | 0.115 | 0.261 |
|  |  | O1(nitr) | -0.260 | 2.023 | 0.2990 | 0.079 | 0.295 |
|  |  | O2(nitr) | -0.263 | 2.015 | 0.3062 | 0.081 | 0.301 |
| 1:1:1 Type **C** | 0.836 | O1 | -0.542 | 1.964 | 0.2379 | 0.086 | 0.356 |
|  |  | O2 | -0.524 | 1.952 | 0.2472 | 0.089 | 0.368 |
|  |  | O1(nitr) | -0.247 | 1.987 | 0.3361 | 0.087 | 0.323 |
|  |  | O2(nitr) | -0.265 | 2.022 | 0.2989 | 0.080 | 0.299 |
| 2:1 Type **D** | 0.653 | O1a | -0.500 | 2.045 | 0.1198 | 0.071 | 0.285 |
|  |  | O4a | -0.425 | 1.932 | 0.1907 | 0.093 | 0.384 |
|  |  | O1b | -0.528 | 1.983 | 0.1282 | 0.081 | 0.333 |
|  |  | O4b | -0.407 | 1.976 | 0.1819 | 0.079 | 0.338 |
| 2:1 Type **E** | 0.728 | O5a | -0.480 | 2.142 | 0.1552 | 0.056 | 0.239 |
|  |  | O6a | -0.475 | 1.850 | 0.3982 | 0.117 | 0.498 |
|  |  | O5b | -0.485 | 2.113 | 0.1584 | 0.060 | 0.255 |
|  |  | O6b | -0.475 | 1.854 | 0.3999 | 0.116 | 0.489 |
| 2:1 Type **F** | 0.514 | N1a | -0.582 | 2.029 | 0.2466 | 0.090 | 0.236 |
|  |  | O4a | -0.255 | 2.215 | 0.1749 | 0.051 | 0.197 |
|  |  | N1b | -0.711 | 2.072 | 0.2318 | 0.082 | 0.218 |
|  |  | O4b | -0.236 | 2.153 | 0.1967 | 0.058 | 0.221 |
| 2:1:1 Type **G** | 0.694 | O1a | -0.486 | 2.145 | 0.1235 | 0.056 | 0.230 |
|  |  | O4a | -0.362 | 2.002 | 0.2021 | 0.077 | 0.314 |
|  |  | O1b | -0.473 | 2.273 | 0.1153 | 0.040 | 0.173 |
|  |  | O4b | -0.415 | 1.968 | 0.1993 | 0.079 | 0.345 |
|  |  | O1(nitr) | -0.329 | 2.150 | 0.1845 | 0.059 | 0.224 |
|  |  | O2(nitr) | -0.234 | 2.367 | 0.1536 | 0.036 | 0.143 |
| 2:1:1 Type **H** | 0.707 | O5a | -0.434 | 2.213 | 0.1237 | 0.046 | 0.205 |
|  |  | O6a | -0.413 | 1.959 | 0.2803 | 0.088 | 0.360 |
|  |  | O5b | -0.409 | 2.466 | 0.0821 | 0.027 | 0.114 |
|  |  | O6b | -0.441 | 1.925 | 0.3037 | 0.094 | 0.395 |
|  |  | O1(nitr) | -0.288 | 2.152 | 0.2121 | 0.058 | 0.226 |
|  |  | O2(nitr) | -0.254 | 2.104 | 0.2411 | 0.065 | 0.226 |
| 2:1:1 Type **I** | 0.471 | N1a | -0.574 | 2.192 | 0.1405 | 0.061 | 0.190 |
|  |  | N1b | -0.677 | 2.059 | 0.2080 | 0.083 | 0.231 |
|  |  | O4b | -0.263 | 2.274 | 0.1614 | 0.044 | 0.178 |
|  |  | O1(nitr) | -0.371 | 1.957 | 0.2830 | 0.089 | 0.352 |

BCP-Bond critical points

**Table S6.** ^1^H NMR calculated isotropic values for colchicine and its complexes (M06/pcS-2).

|  |  | | | |
| --- | --- | --- | --- | --- |
|  | Isotropic values | | | |
| Hydrogen atom | **1** | 1:1:1 Type **A** | 1:1:1 Type **B** | 1:1:1 Type **C** |
| 1H - C4 | 24.67 | 24.48 | 25.00 | 24.38 |
| 2H - C5 | 28.83 | 28.76 | 29.08 | 28.79 |
|  | 29.12 | 28.57 | 28.79 | 29.21 |
| 2H - C6 | 28.98 | 29.42 | 29.24 | 29.14 |
|  | 30.12 | 29.40 | 29.72 | 30.14 |
| 1H - C7 | 26.44 | 27.35 | 25.47 | 26.29 |
| 1H - C8 | 24.04 | 24.12 | 21.91 | 24.33 |
| 1H - C11 | 23.89 | 24.27 | 23.54 | 24.66 |
| 1H - C12 | 24.14 | 23.91 | 23.04 | 24.46 |
| 3H -CH_3_O-1 | 28.94 | 27.44 | 28.60 | 28.34 |
|  | 28.27 | 27.47 | 28.20 | 27.48 |
|  | 27.71 | 27.47 | 26.79 | 28.31 |
| 3H -CH_3_O-2 | 28.18 | 27.15 | 26.57 | 27.43 |
|  | 27.69 | 26.28 | 27.44 | 26.93 |
|  | 27.95 | 27.41 | 28.11 | 25.87 |
| 3H -CH_3_O-3 | 27.78 | 27.49 | 27.48 | 27.75 |
|  | 27.38 | 27.55 | 27.50 | 27.70 |
|  | 27.60 | 27.21 | 27.51 | 27.22 |
| 3H -OCH_3_-10 | 25.98 | 27.67 | 27.10 | 27.63 |
|  | 28.09 | 27.39 | 26.92 | 27.42 |
|  | 27.63 | 27.64 | 27.20 | 27.75 |
| 3H - C14 | 29.45 | 29.90 | 29.25 | 29.79 |
|  | 30.06 | 29.79 | 29.79 | 29.76 |
|  | 29.59 | 30.06 | 29.56 | 29.39 |
| 1H - NH | 25.59 | 25.77 | 24.49 | 25.47 |

Experimental chemical shifts and calculated isotropic values correlated in more than 99%. We used the equation below to calculate chemical shifts.

$$I\times X+Y=CS$$

Were:

I- isotropic

X- -0.9150

Y- 29.0393

CS- calculated value chemical shift

**Table S7.** ^1^H NMR experimental and calculated (M06/pcS-2) chemical shifts data for colchicine and its complexes.

|  | Chemical shifts (ppm) | | | | | |
| --- | --- | --- | --- | --- | --- | --- |
|  | Experimental | | Calculated | | | |
| Hydrogen atom | **1** | Col x Zn(NO_3_)_2_ | **1** | 1:1:1 Type **A** | 1:1:1 Type **B** | 1:1:1 Type **C** |
| 1H - C4 | 6.69 | 6.75 | 6.47 | 6.64 | 6.16 | 6.73 |
| 2H - C5 | 2.32 | 2.2 | 2.66 | 2.72 | 2.43 | 2.70 |
|  | 2.56 | 2.61 | 2.40 | 2.90 | 2.69 | 2.31 |
| 2H - C6 | 1.86 | 1.94 | 2.52 | 2.12 | 2.28 | 2.38 |
|  | 2.09 | 2.18 | 1.48 | 2.14 | 1.85 | 1.46 |
| 1H - C7 | 4.37 | 4.46 | 4.85 | 4.02 | 5.73 | 4.98 |
| 1H - C8 | 7.25 | 7.81 | 7.05 | 6.97 | 8.99 | 6.78 |
| 1H - C11 | 6.93 | 7.5 | 7.18 | 6.83 | 7.50 | 6.47 |
| 1H - C12 | 7.16 | 7.68 | 6.95 | 7.16 | 7.96 | 6.66 |
| 3H -CH_3_O-1 | 3.61 | 3.6 | 2.56 | 3.93 | 2.87 | 3.11 |
|  |  |  | 3.17 | 3.91 | 3.24 | 3.90 |
|  |  |  | 3.69 | 3.90 | 4.53 | 3.14 |
| 3H -CH_3_O-2 | 3.86 | 3.88 | 3.25 | 4.20 | 4.73 | 3.94 |
|  |  |  | 3.70 | 4.99 | 3.93 | 4.40 |
|  |  |  | 3.47 | 3.96 | 3.32 | 5.37 |
| 3H -CH_3_O-3 | 3.83 | 3.85 | 3.62 | 3.89 | 3.90 | 3.65 |
|  |  |  | 3.99 | 3.83 | 3.88 | 3.69 |
|  |  |  | 3.78 | 4.14 | 3.87 | 4.14 |
| 3H -OCH_3_-10 | 3.9 | 4.06 | 5.27 | 3.72 | 4.25 | 3.76 |
|  |  |  | 3.33 | 3.98 | 4.40 | 3.95 |
|  |  |  | 3.76 | 3.75 | 4.15 | 3.65 |
| 3H - C14 | 1.86 | 1.99 | 2.09 | 1.68 | 2.27 | 1.79 |
|  |  |  | 1.54 | 1.78 | 1.78 | 1.81 |
|  |  |  | 1.97 | 1.54 | 1.99 | 2.15 |
| 1H - NH | 7.4 | 7.55 | 5.62 | 5.46 | 6.63 | 5.74 |

**Table S8.** ^1^H NMR calculated isotropic values for colchicine and its complexes (M06/pcS-2).

|  |  | | | |
| --- | --- | --- | --- | --- |
|  | Isotropic values | | | |
| Hydrogen atom | **1** | 1:1:1 Type **A** | 1:1:1 Type **B** | 1:1:1 Type **C** |
| 1H - C4 | 26.04 | 25.83 | 26.42 | 25.71 |
| 2H - C5 | 30.22 | 30.04 | 30.51 | 29.89 |
|  | 30.29 | 29.97 | 30.01 | 30.34 |
| 2H - C6 | 30.12 | 30.46 | 30.47 | 30.13 |
|  | 31.21 | 30.36 | 31.01 | 31.11 |
| 1H - C7 | 27.78 | 28.43 | 26.78 | 27.64 |
| 1H - C8 | 25.06 | 25.30 | 22.77 | 25.29 |
| 1H - C11 | 24.58 | 25.52 | 24.68 | 25.81 |
| 1H - C12 | 25.07 | 25.04 | 24.11 | 25.49 |
| 3H -CH_3_O-1 | 29.87 | 28.54 | 29.64 | 29.82 |
|  | 29.28 | 28.42 | 29.16 | 28.73 |
|  | 28.45 | 28.62 | 27.67 | 29.63 |
| 3H -CH_3_O-2 | 28.94 | 28.55 | 27.44 | 28.68 |
|  | 28.60 | 27.26 | 28.58 | 28.29 |
|  | 28.92 | 28.58 | 29.17 | 26.92 |
| 3H -CH_3_O-3 | 28.93 | 28.68 | 28.68 | 28.69 |
|  | 28.57 | 28.87 | 28.77 | 28.52 |
|  | 28.79 | 28.30 | 28.54 | 28.31 |
| 3H -OCH_3_-10 | 27.28 | 28.80 | 28.27 | 28.65 |
|  | 29.29 | 28.43 | 28.29 | 28.45 |
|  | 28.65 | 28.68 | 28.33 | 28.79 |
| 3H - C14 | 30.67 | 30.92 | 30.59 | 30.93 |
|  | 31.24 | 30.65 | 31.01 | 31.04 |
|  | 30.78 | 31.04 | 30.67 | 30.66 |
| 1H - NH | 27.05 | 27.33 | 26.17 | 26.90 |

Experimental chemical shifts and calculated isotropic values correlated in more than 99%. We used the equation below to calculate chemical shifts.

$$I\times X+Y=CS$$

Were:

I- isotropic

X- -0.9311

Y- 30.5524

CS- calculated value chemical shift

**Table S9.** ^1^H NMR experimental and calculated (M06/SDD) chemical shifts data for colchicine and its complexes.

|  | Chemical shifts (ppm) | | | | | |
| --- | --- | --- | --- | --- | --- | --- |
|  | Experimental | | Calculated | | | |
| Hydrogen atom | **1** | Col x Zn(NO_3_)_2_ | **1** | 1:1:1 Type **A** | 1:1:1 Type **B** | 1:1:1 Type **C** |
| 1H - C4 | 6.69 | 6.75 | 6.31 | 6.50 | 5.95 | 6.62 |
| 2H - C5 | 2.32 | 2.2 | 2.42 | 2.58 | 2.15 | 2.72 |
|  | 2.56 | 2.61 | 2.35 | 2.64 | 2.61 | 2.30 |
| 2H - C6 | 1.86 | 1.94 | 2.51 | 2.19 | 2.18 | 2.50 |
|  | 2.09 | 2.18 | 1.49 | 2.29 | 1.68 | 1.58 |
| 1H - C7 | 4.37 | 4.46 | 4.68 | 4.08 | 5.61 | 4.82 |
| 1H - C8 | 7.25 | 7.81 | 7.21 | 6.99 | 9.35 | 7.00 |
| 1H - C11 | 6.93 | 7.5 | 7.67 | 6.79 | 7.57 | 6.52 |
| 1H - C12 | 7.16 | 7.68 | 7.21 | 7.24 | 8.10 | 6.82 |
| 3H -CH_3_O-1 | 3.61 | 3.6 | 2.74 | 3.97 | 2.95 | 2.79 |
|  |  |  | 3.28 | 4.09 | 3.40 | 3.80 |
|  |  |  | 4.06 | 3.90 | 4.79 | 2.96 |
| 3H -CH_3_O-2 | 3.86 | 3.88 | 3.61 | 3.97 | 5.00 | 3.85 |
|  |  |  | 3.92 | 5.17 | 3.94 | 4.21 |
|  |  |  | 3.62 | 3.94 | 3.39 | 5.49 |
| 3H -CH_3_O-3 | 3.83 | 3.85 | 3.61 | 3.84 | 3.85 | 3.84 |
|  |  |  | 3.95 | 3.67 | 3.76 | 4.00 |
|  |  |  | 3.74 | 4.20 | 3.98 | 4.19 |
| 3H -OCH_3_-10 | 3.9 | 4.06 | 5.15 | 3.73 | 4.23 | 3.88 |
|  |  |  | 3.28 | 4.08 | 4.21 | 4.06 |
|  |  |  | 3.87 | 3.85 | 4.18 | 3.74 |
| 3H - C14 | 1.86 | 1.99 | 2.00 | 1.77 | 2.07 | 1.76 |
|  |  |  | 1.46 | 2.01 | 1.68 | 1.65 |
|  |  |  | 1.89 | 1.65 | 1.99 | 2.00 |
| 1H - NH | 7.4 | 7.55 | 5.37 | 5.10 | 6.19 | 5.50 |

**Table S10.** ^13^C NMR calculated isotropic values for colchicine and its complexes (M06/pcS-2).

|  | Isotropic values | | | |  |
| --- | --- | --- | --- | --- | --- |
| Atom C | Calculated | | | | |
|  | 1 | 1:1:1 Type D | 1:1:1 Type E | 1:1:1 Type F | |
| C1 | 2.87 | 7.89 | 4.00 | 8.82 | |
| C1a | 37.72 | 32.49 | 39.59 | 42.44 | |
| C2 | 18.85 | 23.71 | 19.79 | 26.85 | |
| C3 | 2.42 | 1.30 | -0.10 | 7.59 | |
| C4 | 58.93 | 50.71 | 58.46 | 54.86 | |
| C4a | 15.78 | 16.66 | 16.19 | 10.44 | |
| C5 | 143.02 | 143.92 | 142.37 | 143.23 | |
| C6 | 138.49 | 134.07 | 138.05 | 139.90 | |
| C7 | 110.61 | 102.67 | 114.62 | 112.38 | |
| C7a | 10.21 | 16.52 | -5.48 | 12.11 | |
| C8 | 17.05 | 21.52 | 17.24 | 23.90 | |
| C9 | -24.83 | -24.02 | -13.14 | -30.06 | |
| C10 | -7.90 | -9.23 | -0.21 | -7.08 | |
| C11 | 43.15 | 52.36 | 43.13 | 54.40 | |
| C12 | 16.97 | 25.62 | 9.93 | 20.80 | |
| C12a | 23.95 | 27.08 | 11.69 | 29.45 | |
| OCH_3_(1) | 113.95 | 111.69 | 111.54 | 111.45 | |
| OCH_3_(2) | 108.11 | 110.12 | 107.23 | 110.64 | |
| OCH_3_(3) | 106.46 | 99.23 | 106.37 | 94.26 | |
| OCH_3_(10) | 107.02 | 101.20 | 106.80 | 94.84 | |
| C=O(CH_3_) | -17.54 | -25.47 | -20.70 | -18.42 | |
| CH_3_(C=O) | 150.56 | 147.59 | 151.39 | 151.69 | |

Experimental chemical shifts and calculated isotropic values correlated in more than 99%. We used the equation below to calculate chemical shifts.

$$I\times X+Y=CS$$

Were:

I- isotropic

X- -0.8849

Y- 156.8398

CS- calculated value chemical shift

**Table S11.** ^13^C NMR experimental and calculated (mM06/pcS-2) chemical shifts data for colchicine and its complexes.

|  | Chemical shifts (ppm) | | | | | |
| --- | --- | --- | --- | --- | --- | --- |
| Atom C | Experimental | | Calculated | | | |
|  | **1** | **Col Zn(NO_3_)_2_** | 1 | 1:1:1 Type D | 1:1:1 Type E | 1:1:1 Type F |
| C1 | 151.72 | 151.56 | 154.30 | 149.86 | 153.30 | 149.03 |
| C1a | 126.57 | 125.29 | 123.46 | 128.09 | 121.80 | 119.28 |
| C2 | 142.12 | 142.14 | 140.16 | 135.86 | 139.32 | 133.08 |
| C3 | 154.41 | 157.69 | 154.70 | 155.69 | 156.93 | 150.12 |
| C4 | 108.52 | 108.79 | 104.69 | 111.96 | 105.10 | 108.29 |
| C4a | 136.84 | 141.92 | 142.88 | 142.10 | 142.51 | 147.60 |
| C5 | 30.27 | 29.71 | 30.27 | 29.47 | 30.85 | 30.08 |
| C6 | 36.84 | 37.15 | 34.27 | 38.19 | 34.67 | 33.03 |
| C7 | 52.95 | 54.22 | 58.95 | 65.98 | 55.40 | 57.38 |
| C7a | 152.01 | 155.32 | 147.81 | 142.22 | 161.69 | 146.12 |
| C8 | 131.32 | 131.43 | 141.75 | 137.79 | 141.59 | 135.69 |
| C9 | 179.63 | 178.48 | 178.81 | 178.10 | 168.47 | 183.44 |
| C10 | 164.88 | 163.14 | 163.83 | 165.01 | 157.02 | 163.10 |
| C11 | 112.98 | 118.9 | 118.65 | 110.50 | 118.67 | 108.70 |
| C12 | 136.66 | 140.13 | 141.82 | 134.16 | 148.05 | 138.43 |
| C12a | 135.57 | 135.73 | 135.65 | 132.87 | 146.49 | 130.78 |
| OCH_3_(1) | 61.61 | 61.95 | 62.63 | 69.02 | 62.70 | 73.42 |
| OCH_3_(2) | 61.39 | 61.47 | 62.13 | 67.28 | 62.32 | 72.91 |
| OCH_3_(3) | 56.58 | 56.67 | 55.99 | 57.99 | 58.13 | 58.21 |
| OCH_3_(10) | 56.76 | 58.33 | 61.16 | 59.38 | 61.94 | 58.93 |
| C=O(CH_3_) | 170.04 | 172.16 | 172.36 | 179.38 | 175.16 | 173.14 |
| CH_3_(C=O) | 22.72 | 22.71 | 23.59 | 26.22 | 22.86 | 22.59 |

**Table S12.** ^13^C NMR calculated isotropic values for colchicine and its complexes (M06/SDD).

|  | Isotropic values | | | |  |
| --- | --- | --- | --- | --- | --- |
| Atom C | Calculated | | | | |
|  | 1 | 1:1:1 Type D | 1:1:1 Type E | 1:1:1 Type F | |
| C1 | 26.19 | 33.84 | 28.10 | 35.93 | |
| C1a | 57.57 | 52.18 | 58.20 | 60.93 | |
| C2 | 40.26 | 46.60 | 41.74 | 50.40 | |
| C3 | 26.76 | 26.15 | 24.85 | 32.17 | |
| C4 | 73.35 | 66.65 | 74.11 | 70.64 | |
| C4a | 39.91 | 38.28 | 40.32 | 34.04 | |
| C5 | 154.18 | 156.26 | 154.63 | 154.38 | |
| C6 | 151.93 | 146.15 | 150.01 | 152.81 | |
| C7 | 123.42 | 116.11 | 128.98 | 124.85 | |
| C7a | 34.00 | 40.52 | 20.25 | 35.32 | |
| C8 | 33.00 | 39.32 | 30.26 | 39.81 | |
| C9 | -0.93 | -0.28 | 10.43 | -5.58 | |
| C10 | 17.79 | 15.28 | 23.53 | 18.32 | |
| C11 | 61.49 | 70.55 | 62.91 | 72.84 | |
| C12 | 36.97 | 46.20 | 29.44 | 42.94 | |
| C12a | 47.04 | 48.94 | 36.47 | 51.63 | |
| OCH_3_(1) | 118.27 | 112.34 | 119.23 | 111.16 | |
| OCH_3_(2) | 119.02 | 114.37 | 119.98 | 112.81 | |
| OCH_3_(3) | 124.58 | 122.55 | 122.83 | 122.55 | |
| OCH_3_(10) | 120.21 | 121.50 | 119.44 | 121.78 | |
| C=O(CH_3_) | 8.03 | 0.72 | 7.28 | 7.94 | |
| CH_3_(C=O) | 158.56 | 156.65 | 159.30 | 159.62 | |

Experimental chemical shifts and calculated isotropic values correlated in more than 99%. We used the equation below to calculate chemical shifts.

$$I\times X+Y=CS$$

Were:

I- isotropic

X- -0.9903

Y- 180.0189

CS- calculated value chemical shift

**Table S13.** ^13^C NMR experimental and calculated (mM06/SDD) chemical shifts data for colchicine and its complexes.

|  | Chemical shifts (ppm) | | | | | |
| --- | --- | --- | --- | --- | --- | --- |
| Atom C | Experimental | | Calculated | | | |
|  | **1** | **Col Zn(NO_3_)_2_** | 1 | 1:1:1 Type D | 1:1:1 Type E | 1:1:1 Type F |
| C1 | 151.72 | 151.56 | 154.08 | 146.50 | 152.19 | 144.44 |
| C1a | 126.57 | 125.29 | 123.00 | 128.34 | 122.38 | 119.68 |
| C2 | 142.12 | 142.14 | 140.15 | 133.87 | 138.69 | 130.11 |
| C3 | 154.41 | 157.69 | 153.52 | 154.12 | 155.41 | 148.16 |
| C4 | 108.52 | 108.79 | 107.39 | 114.01 | 106.63 | 110.07 |
| C4a | 136.84 | 141.92 | 140.50 | 142.11 | 140.09 | 146.31 |
| C5 | 30.27 | 29.71 | 27.34 | 25.28 | 26.89 | 27.14 |
| C6 | 36.84 | 37.15 | 29.56 | 35.29 | 31.47 | 28.69 |
| C7 | 52.95 | 54.22 | 57.80 | 65.04 | 52.29 | 56.39 |
| C7a | 152.01 | 155.32 | 146.35 | 139.90 | 159.96 | 145.05 |
| C8 | 131.32 | 131.43 | 147.34 | 141.08 | 150.05 | 140.60 |
| C9 | 179.63 | 178.48 | 180.94 | 180.29 | 169.69 | 185.54 |
| C10 | 164.88 | 163.14 | 162.40 | 164.88 | 156.72 | 161.87 |
| C11 | 112.98 | 118.9 | 119.13 | 110.16 | 117.72 | 107.89 |
| C12 | 136.66 | 140.13 | 143.41 | 134.27 | 150.87 | 137.49 |
| C12a | 135.57 | 135.73 | 133.44 | 131.56 | 143.90 | 128.89 |
| OCH_3_(1) | 61.61 | 61.95 | 62.90 | 68.77 | 61.95 | 69.94 |
| OCH_3_(2) | 61.39 | 61.47 | 62.16 | 66.76 | 61.21 | 68.31 |
| OCH_3_(3) | 56.58 | 56.67 | 56.65 | 58.66 | 58.38 | 58.66 |
| OCH_3_(10) | 56.76 | 58.33 | 60.97 | 59.70 | 61.74 | 59.43 |
| C=O(CH_3_) | 170.04 | 172.16 | 172.07 | 179.31 | 172.81 | 172.16 |
| CH_3_(C=O) | 22.72 | 22.71 | 23.00 | 24.89 | 22.27 | 21.96 |

**Table S14.** Atomic coordinates of seven-membered ring in most energetically favoured 1:1:1 stoichiometry colchicine complex with zinc cation.

|  | Atomic coordinates | | |
| --- | --- | --- | --- |
|  | 1:1:1 Stoichiometry Type A complex | | |
| Atom | x | y | z |
| c7 | 1.449 | 1.954 | 1.779 |
| c7a | 2.193 | 0.953 | 0.888 |
| c12a | 1.855 | 0.864 | -0.519 |
| c1a | 0.513 | 1.382 | -0.890 |
| c4a | 0.156 | 2.732 | -0.706 |
| c5 | 1.238 | 3.663 | -0.228 |
| c6 | 1.596 | 3.410 | 1.263 |

**Table S15.** Atomic coordinates of both seven-membered rings in most energetically favoured 2:1 stoichiometry colchicine complex with zinc cation.

|  | Atomic coordinates | | | | | |
| --- | --- | --- | --- | --- | --- | --- |
|  | 2:1 Stoichiometry Type E complex | | | | | |
| Atom | x | y | z | x | y | z |
| c7 | -5.942 | 2.596 | -0.593 | 5.871 | -2.410 | 0.700 |
| c7a | -5.368 | 1.207 | -0.199 | 5.002 | -1.297 | 0.097 |
| c12a | -6.120 | 0.081 | 0.284 | 5.649 | -0.176 | -0.522 |
| c1a | -7.584 | -0.115 | 0.130 | 7.115 | -0.196 | -0.709 |
| c4a | -8.559 | 0.868 | 0.413 | 7.754 | -1.326 | -1.266 |
| c5 | -8.149 | 2.293 | 0.654 | 6.942 | -2.540 | -1.643 |
| c6 | -7.453 | 2.801 | -0.623 | 6.464 | -3.318 | -0.399 |

**Table S16.** Atomic coordinates of both seven-membered rings in most energetically favoured 2:1:1 stoichiometry colchicine complex with zinc cation.

|  | Atomic coordinates | | | | | |
| --- | --- | --- | --- | --- | --- | --- |
|  | 2:1:1 Stoichiometry Type H complex | | | | | |
| Atom | x | y | z | x | y | z |
| c7 | -6.010 | -0.006 | 2.351 | 5.264 | -1.984 | 1.308 |
| c7a | -5.140 | 0.238 | 1.110 | 4.747 | -0.875 | 0.379 |
| c12a | -5.766 | 0.148 | -0.182 | 5.665 | -0.275 | -0.546 |
| c1a | -7.246 | -0.008 | -0.251 | 7.049 | -0.810 | -0.643 |
| c4a | -8.089 | 0.868 | 0.463 | 7.271 | -2.189 | -0.822 |
| c5 | -7.487 | 1.896 | 1.388 | 6.095 | -3.133 | -0.857 |
| c6 | -6.892 | 1.228 | 2.650 | 5.458 | -3.308 | 0.540 |


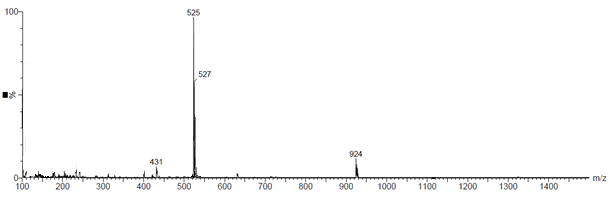


**Figure S1.** ESI MS mass spectra of colchicine complexes with zinc nitrate.

**Figure S2.** Experimental IR spectra of colchicine in KBr pellet.

**Figure S3.** Experimental IR spectra of colchicine complex in KBr pellet.

**Figure S4.** Experimental IR spectra of colchicine in nujol.

**Figure S5.** Experimental IR spectra of colchicine complex in nujol.

**Figure S6.** Experimental IR spectra of colchicine in CD_3_CN.

**Figure S7.** Experimental IR spectra of colchicine complex in CD_3_CN.


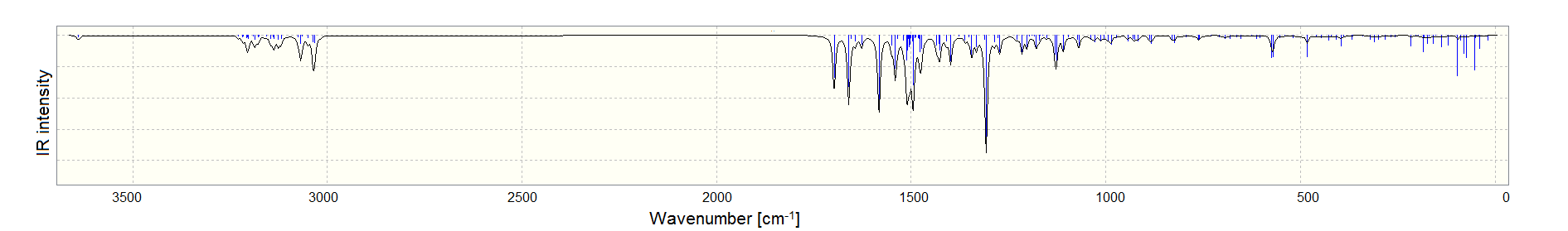


**Figure S8.** Calculated IR spectra of colchicine in vacuum.


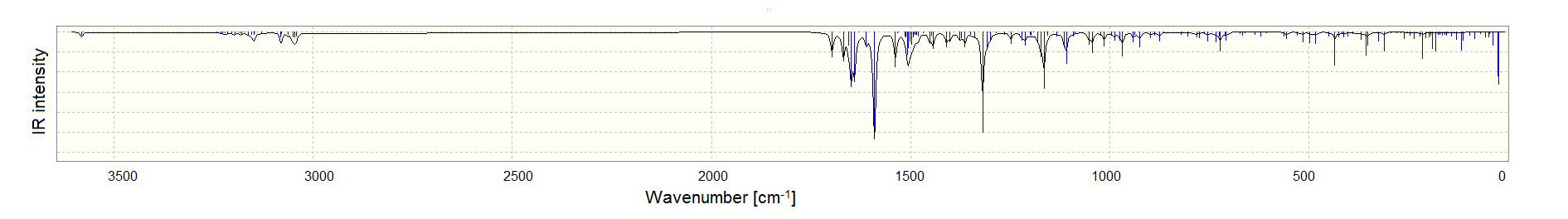


**Figure S9.** Calculated IR spectra of colchicine 1:1:1 stoichiometry complex Type **A** in vacuum.


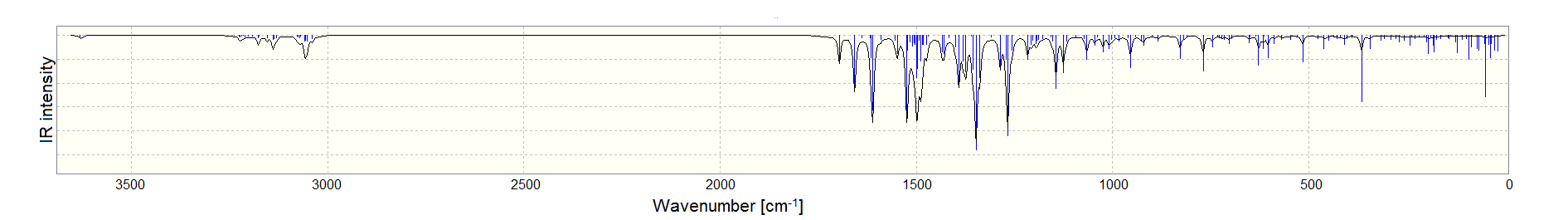


**Figure S10.** Calculated IR spectra of colchicine 1:1:1 stoichiometry complex Type **B** in vacuum.


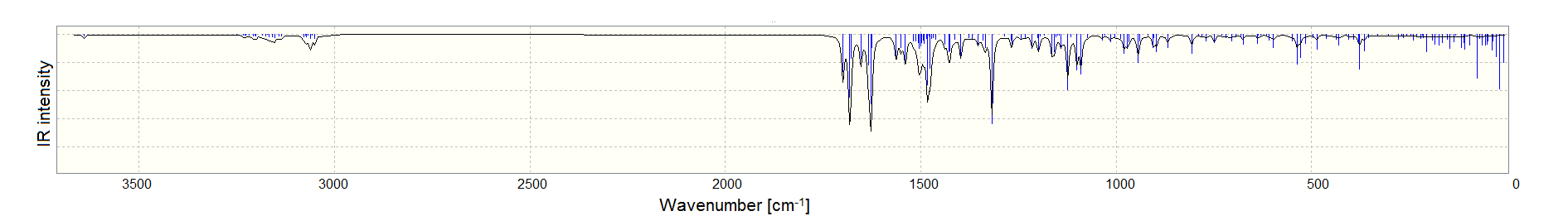


**Figure S11.** Calculated IR spectra of colchicine 1:1:1 stoichiometry complex Type **C** in vacuum.


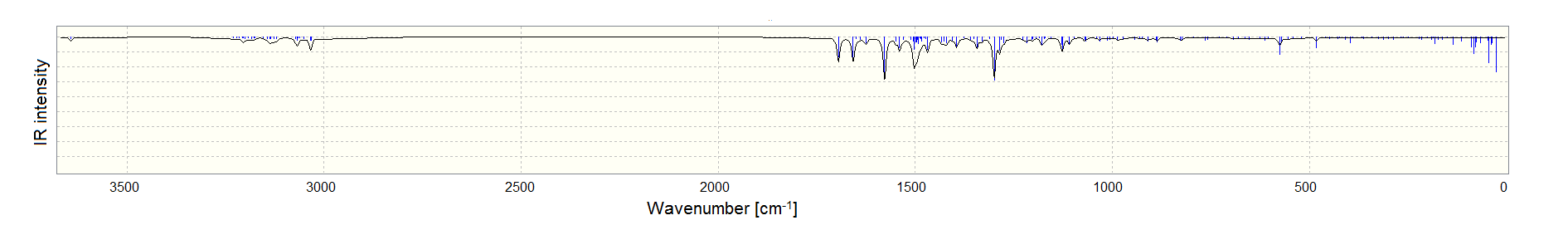


**Figure S12.** Calculated IR spectra of colchicine in nujol.


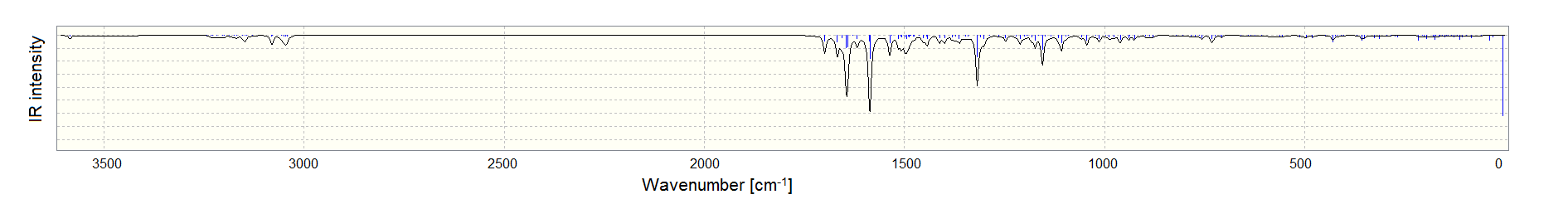


**Figure S13.** Calculated IR spectra of colchicine complex 1:1:1 stoichiometry complex Type **A** in nujol.


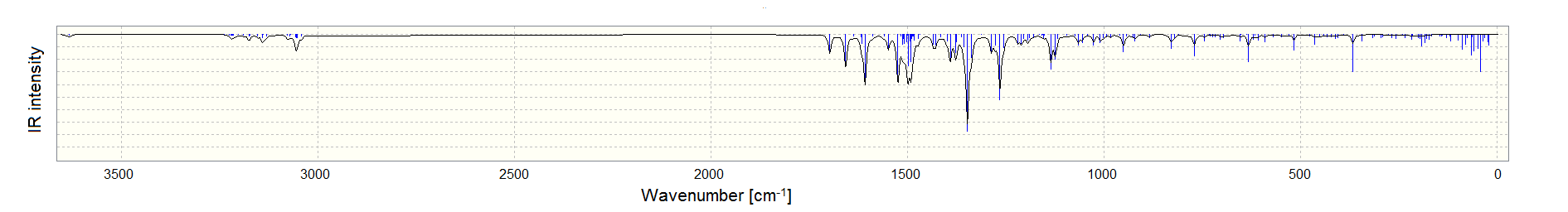


**Figure S14.** Calculated IR spectra of colchicine complex 1:1:1 stoichiometry complex Type **B** in nujol.


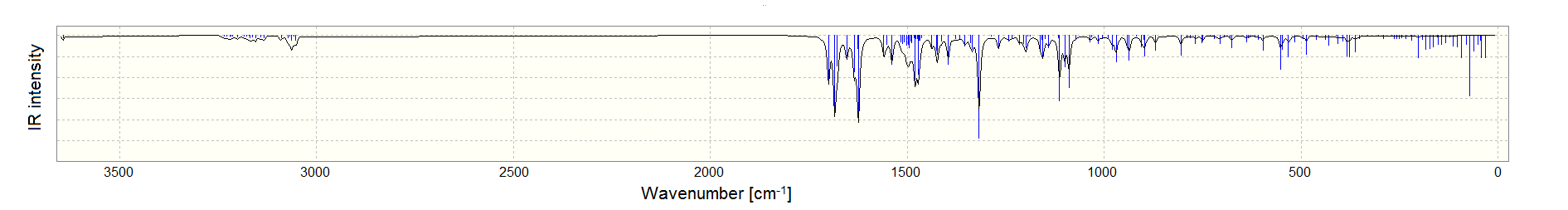


**Figure S15.** Calculated IR spectra of colchicine complex 1:1:1 stoichiometry complex Type **C** in nujol.


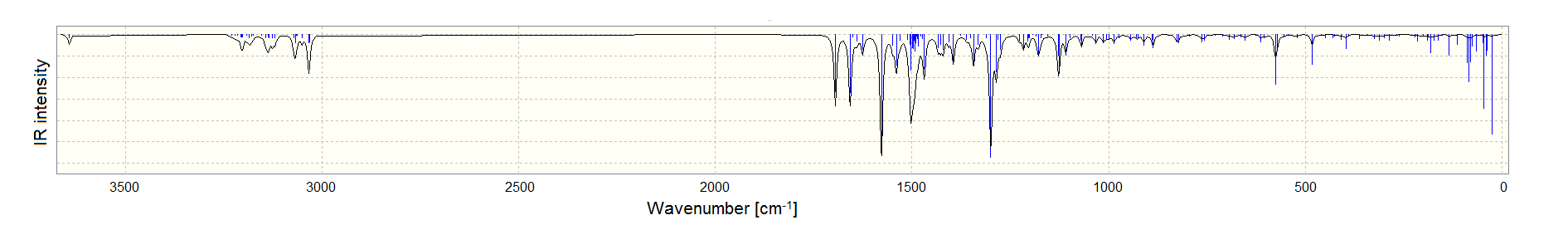


**Figure S16.** Calculated IR spectra of colchicine in CD_3_CN.


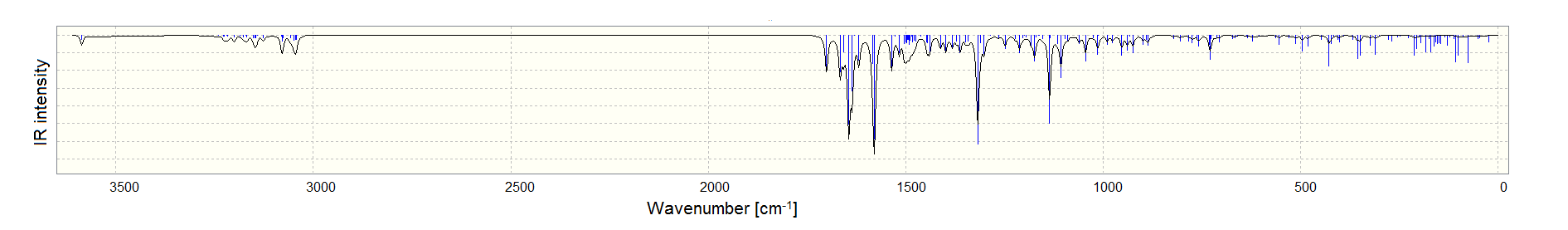


**Figure S17.** Calculated IR spectra of colchicine 1:1:1 stoichiometry complex Type **A** complex in CD_3_CN.


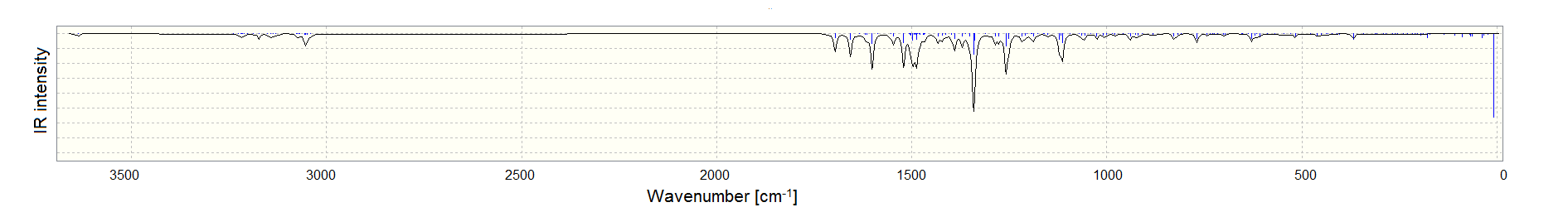


**Figure S18.** Calculated IR spectra of colchicine 1:1:1 stoichiometry complex Type **B** complex in CD_3_CN.


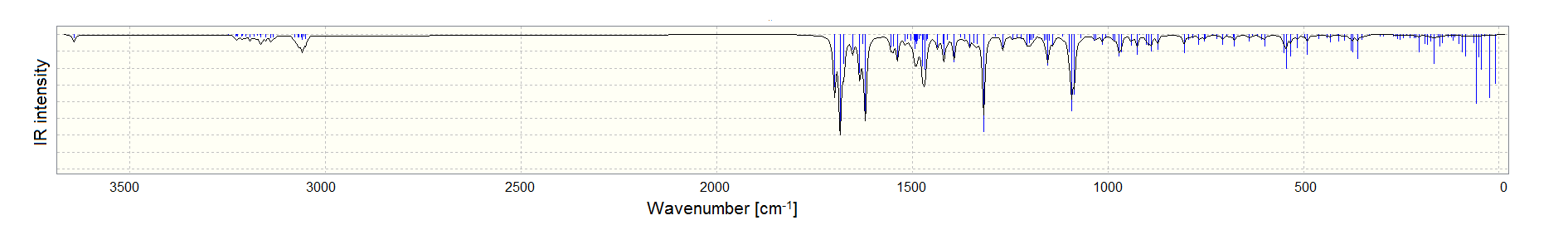


**Figure S19.** Calculated IR spectra of colchicine 1:1:1 stoichiometry complex Type **C** complex in CD_3_CN.


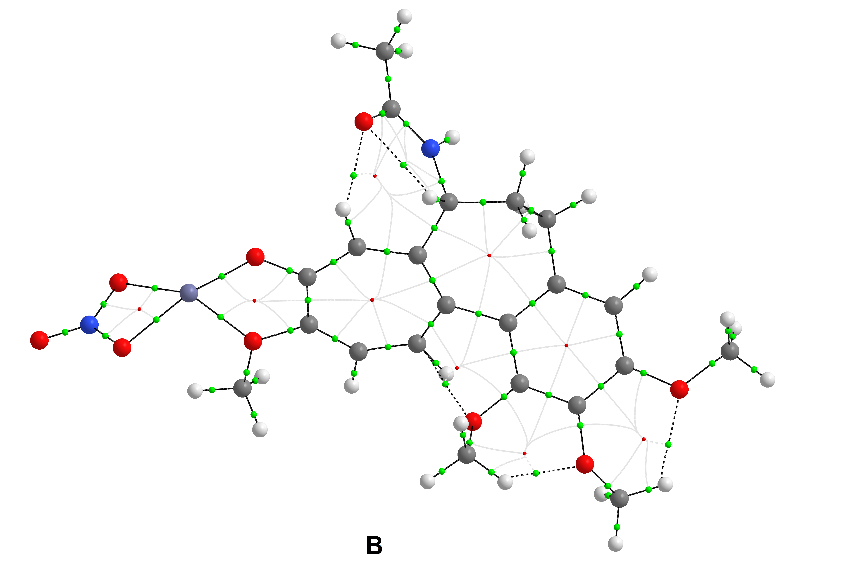


**Figure S20.** Bonds paths (black) and bonds critical points (green) of 1:1:1 stoichiometry Type **B** complex of colchicine with Zn^2+^.


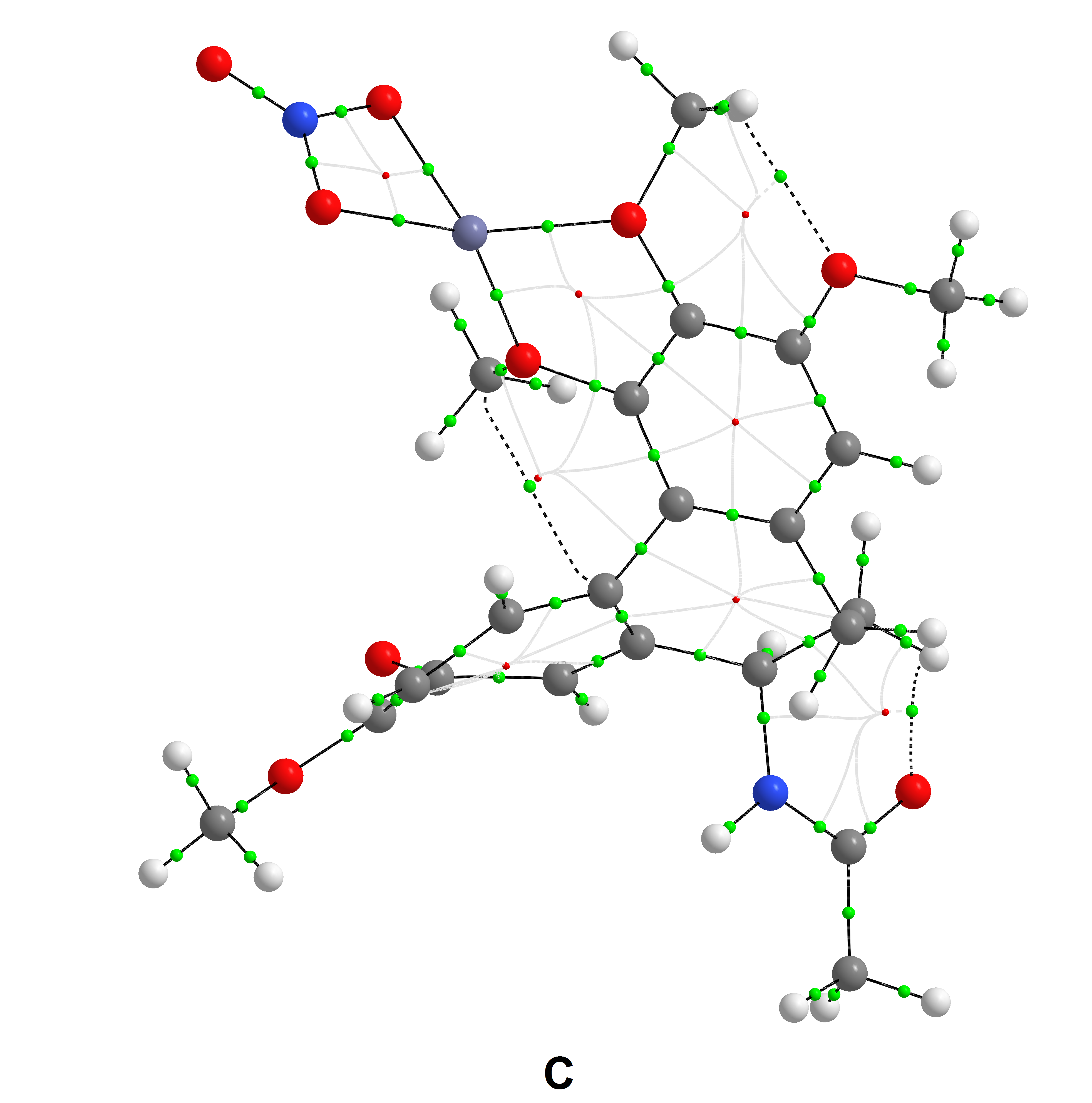


**Figure S21.** Bonds paths (black) and bonds critical points (green) of 1:1:1 stoichiometry Type **C** complex of colchicine with Zn^2+^.


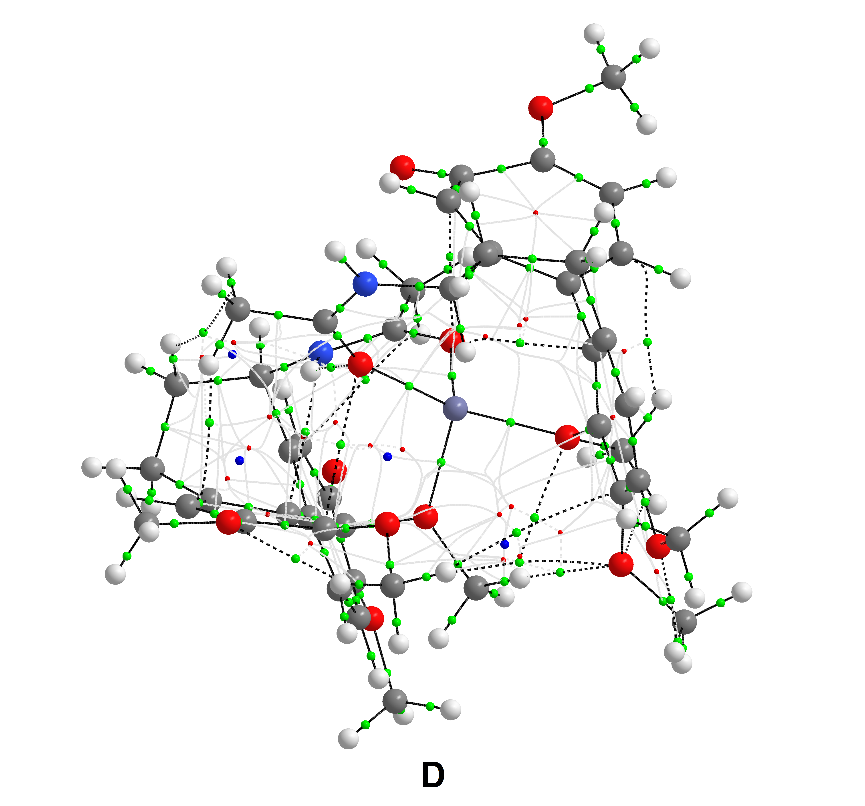


**Figure S22.** Bonds paths (black) and bonds critical points (green) of 2:1 stoichiometry Type **B** complex of colchicine with Zn^2+^.


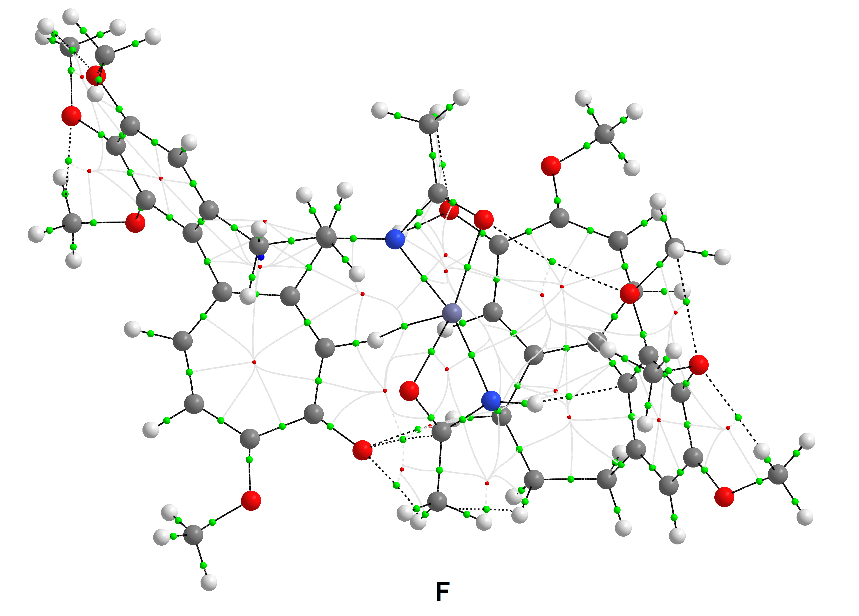


**Figure S23.** Bonds paths (black) and bonds critical points (green) of 2:1 stoichiometry Type **F** complex of colchicine with Zn^2+^.


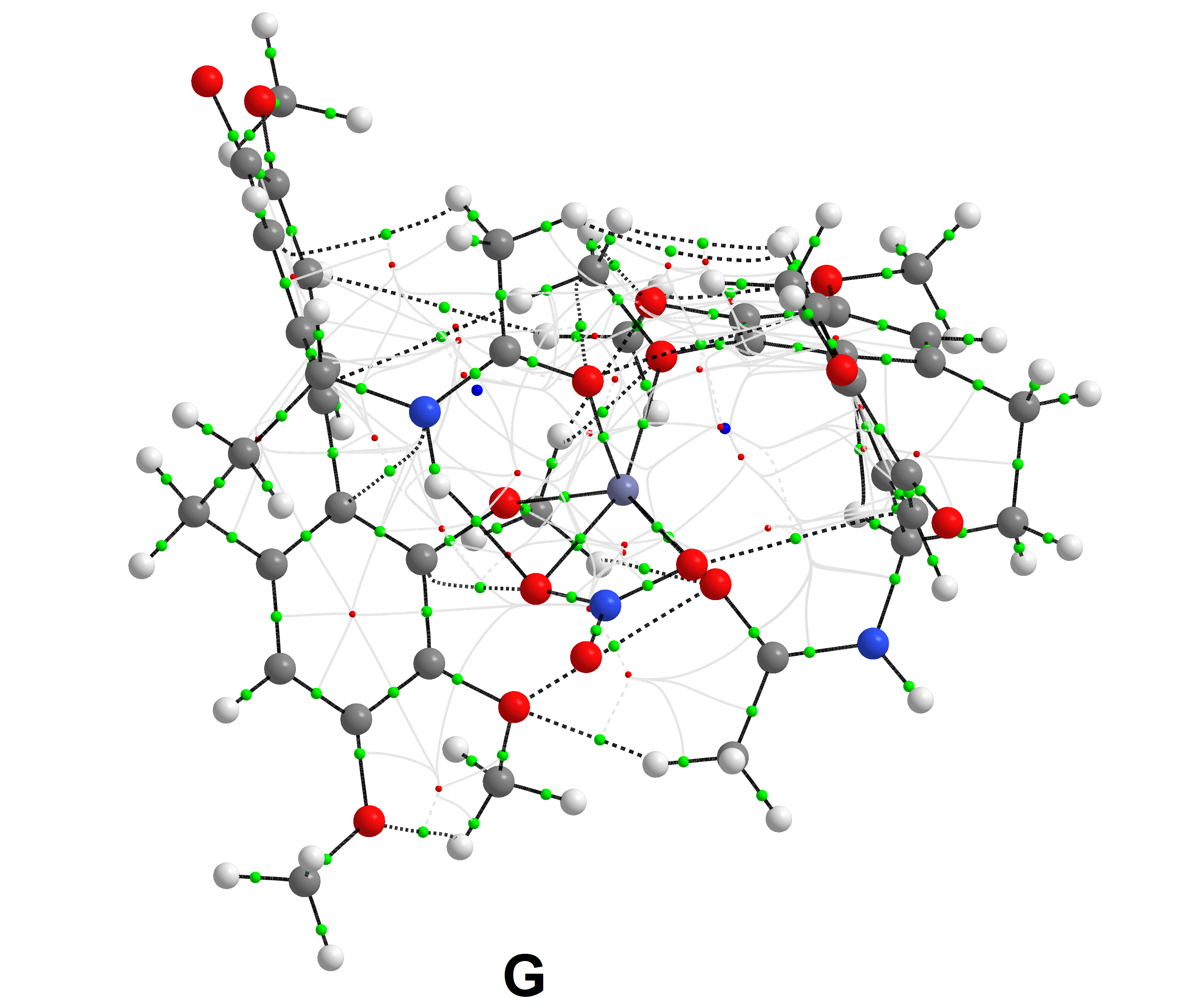


**Figure S24.** Bonds paths (black) and bonds critical points (green) of 2:1 stoichiometry Type **G** complex of colchicine with Zn^2+^.


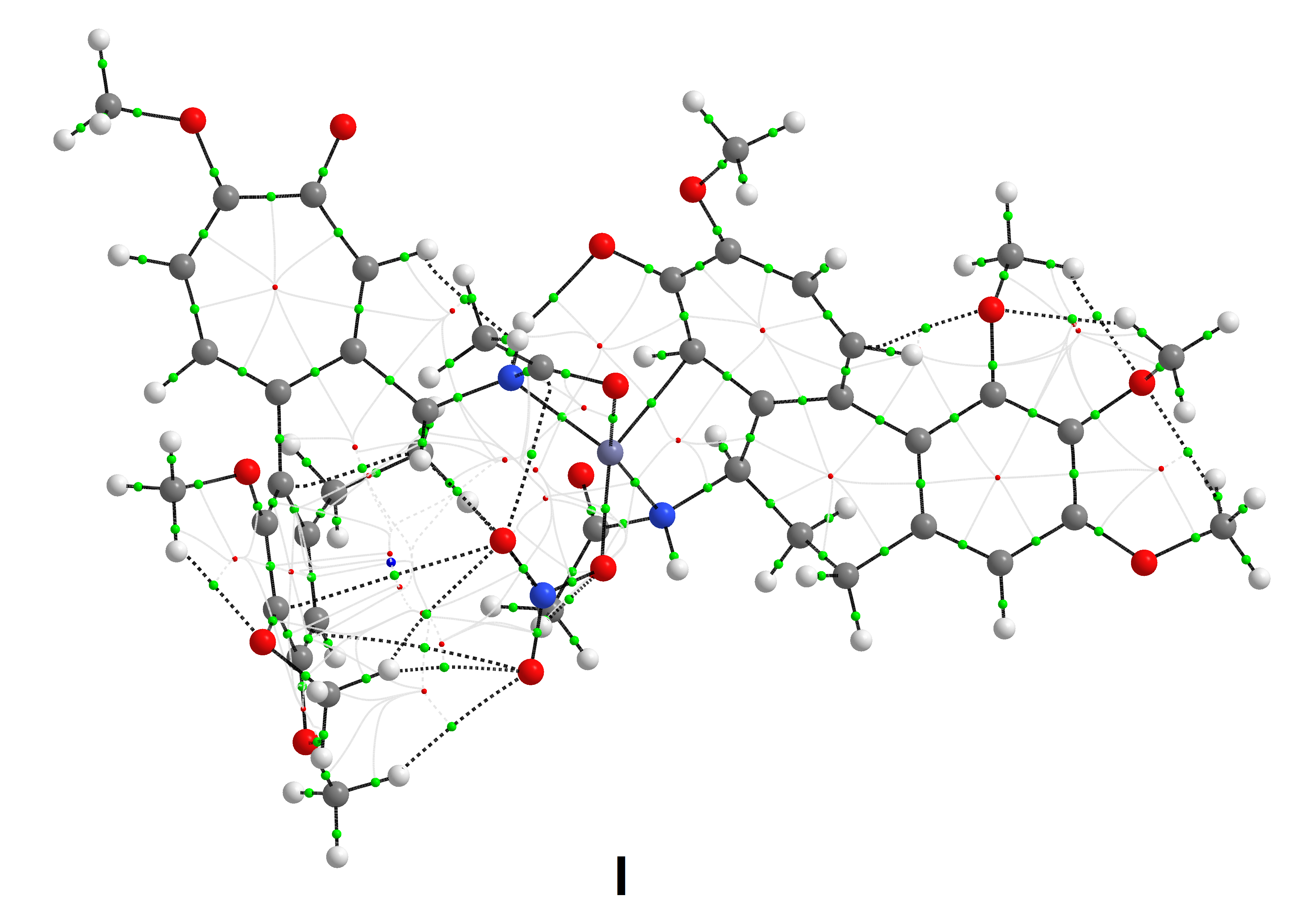


**Figure S25.** Bonds paths (black) and bonds critical points (green) of 2:1:1 stoichiometry Type **I** complex of colchicine with Zn^2+^.
